# BALOs Improved Gut Microbiota Health in Postlarval Shrimp (*Litopenaeus vannamei*) After Being Subjected to Salinity Reduction Treatment

**DOI:** 10.3389/fmicb.2020.01296

**Published:** 2020-06-30

**Authors:** Qingqing Cao, Farhana Najnine, Hongcao Han, Bing Wu, Junpeng Cai

**Affiliations:** ^1^School of Food Science and Engineering, South China University of Technology, Guangzhou, China; ^2^School of Bioscience and Bioengineering, South China University of Technology, Guangzhou, China; ^3^Modern Analysis Centre, South China University of Technology, Guangzhou, China

**Keywords:** *Bdellovibrio* and like organisms, white shrimp postlarvae, microbiota health, Shannon index, mechanism, salinity reduction treatment, *Vibrio*, *Gammaproteobacteria*

## Abstract

White shrimp, *Litopenaeus vannamei*, is a widely farmed species. In China, shrimp postlarvae (PL) are frequently subjected to salinity reduction treatment to meet end growers’ needs. However, although this treatment effectively reduces vibrio counts, its impact on gut microbiota health is still unknown. In this study, we applied a euryhaline strain of BALOs, BDN-1F2 (BD), and *Bacillus subtilis* (SD) to the rearing of second-generation shrimp PL after salinity reduction treatment so as to determine if they could impact PL gut microbiota by using high-throughput sequencing analysis. Results show that PL gut microbiota, both compositionally and functionally, have been badly wrecked after salinity reduction treatment with the generally recognized as opportunistic pathogens *Gammaproteobacteria* being the only dominant class at day 1 of test, viz., 99.43, 85.61, and 83.28% in BD, SD, and control (CD) groups, respectively. At day 7, *Gammaproteobacteria* was still the only dominant class in the SD and CD groups with relative abundance of 99.77 and 99.87% correspondingly, whereas in the BD group, its value dropped to 8.44%. Regarding biodiversity parameter the Shannon index, over the 7-day test period, while the SD group was unchanged (0.98–0.93), the CD group dropped to 0.94 from 2.94, and the BD group was raised to 7.14 from 0.93. Functionally, compared to control, the SD group displayed similar strength of various predicted community functions, but the BD group had hugely enhanced its various capabilities (*p* < 0.05). These results demonstrated that the addition of BDN-1F2 had exceedingly improved PL gut microbiota health by raising its biodiversities and strengthening its functionalities. On reviewing data derived from this as well as relevant studies, a Shannon index cutoff value was tentatively suggested so as to differentiate microbiota-healthy PL7-15 from the unhealthy ones. Furthermore, a conceptual mechanism of BALOs in the rectification/improvement of the microbial community health has also been proposed.

## Introduction

White shrimp, *Litopenaeus vannamei* (Boone, 1931), is one of the most widely grown and commercially successful shrimp/crustacean species around the world. China is one of the world’s leading shrimp producers. Of the 4,155,827 metric tons of shrimp production in 2016, China accounted for 38.8% (Tacon, 2020).

With the expansion of intensive shrimp farming, disease outbreaks have become more widespread than ever before. A wide range of infectious agents, including viral, rickettsial, bacterial, fungal, protistan, and metazoan ones, have been reported (Lightner and Redman, 1998), making disease prevention extremely difficult if not possible. Thus, in order to produce fast-growing and all-round healthier larvae/postlarvae (PL), which are also free from some specific pathogens (SPF), such as white spot syndrome virus and taura syndrome virus (Chen W.-Y. et al., 2017), some Chinese shrimp breeders are looking abroad and import shrimp brood stocks from overseas. The offspring produced by imported oversea brood stocks are called first-generation shrimp larvae/PL. Once these offspring grow up, they might be selected by some breeders to produce larvae/PL, which we call second-generation larvae/PL. Compared to the first-generation larvae/PL that are believed to be growing faster and are better resistant to diseases but are less adapted to the local environments, second-generation larvae/PL are believed to be more adapted to the local climate, albeit with slower growth and weaker disease resistance (Xie et al., 2019). Both types of PL have their own preferences among Chinese shrimp growers.

As vibrios are a group of well-recognized opportunistic pathogens that could cause vibriosis in shrimp (Saulnier et al., 2000), a common practice in many PL production farms in China is to subject PL of 4–5 days old (PL4-5) in 7–10 days’ time to, first, a gradual reduction in salinity from around 20‰ to 4‰–5‰ or even zero, then back to various salinities to suit shrimp growers’ needs. By so doing, the number of vibrios is reduced, and green vibrios, which are viewed as more virulent than their yellow counterparts, could be lowered to undetected levels or even totally eliminated. The effect of this process was demonstrated by Liu et al. (2004), who reported that PL shrimp subjected to a gradual salinity reduction resulted in lower counts of vibrios within their bodies. From a salinity of 14.36‰ to 0‰ over a 7-day period, vibrio numbers were down from 2.3 × 10^4^ colony-forming units (CFU) mg^–1^ (of 71.6% of total bacterial counts) to 5.6 × 10^2^ CFU mg^–1^ (of 39.6% of total bacterial counts). Heterogenic bacterial numbers were down from 3.24 × 10^4^ CFU mg^–1^ to 1.43 × 10^3^ CFU mg^–1^. They also noted that, by lowering salinities, PL shrimp luminous vibriosis could be contained and eventually eliminated in an outbreak. Nevertheless, they pointed out that, though this salinity reduction treatment enabled PL to carry fewer vibrios, whether it could really give rise to healthier PL is still an open question. It is particularly relevant when considering the situation that, in recent years, a quite common phenomenon has been noted in many shrimp grow-out farms in China; that is, once PL8-10 were stocked in grow-out ponds, they frequently experienced symptoms of disease, such as white feces and even mortalities within 30 days, some as early as 8–10 days (Zhao, 2014). Could this have anything to do with salinity reduction treatment that reduces or eliminates vibrios in PL while unknowingly wrecking the gut microbiota and making it less adaptable to various environments and/or more vulnerable to (potential) pathogenic attacks? If so, could probiotics, such as *Bacillus* and BALOs, be used to rectify it?

Gut microbiota has been shown to serve as a virtual endocrine organ to influence host metabolism and body composition and to prevent pathogen invasions (Clarke et al., 2014). “Changes in shrimp intestinal bacterial communities occurred in parallel with changes in disease severity” (Xiong et al., 2015b). Thus, it is clear that the balanced microbial community structures are crucial in maintaining health and preventing diseases (Semova et al., 2012; Coretti et al., 2017). Nevertheless, in the laviculture of various aquatic species, as “detrimental larvae-microbe interactions are a main reason for poor viability and quality in larval rearing” (Vadstein et al., 2018), many means have, therefore, been employed to counteract this detrimental interaction, among them including the antibiotic prophylactic method (De Schryver and Vadstein, 2014) and the abovementioned salinity reduction treatment (Liu et al., 2004). Although the antibiotic prophylactic method had been shown to cause an imbalance of the microbial community, such as reduction of the Shannon diversity index (Zeng et al., 2019), the effect of the latter on gut microbiota was still unknown.

*Bdellovibrio* and like organisms (BALOs) are a group of tiny sized (ca. 1/10th of the size of *Escherichia coli*) bacteria, naturally existing in terrestrial and/or aquatic ecosystems as well as in the intestine of organisms such as eels (Zhang et al., 2009), white shrimp (Huang et al., 2016), and humans (Iebba et al., 2013). Taxonomically, they are very diverse but with a common feature, that is, killing another bacterium by means of periplasmic, epibiotic, or wolfpack attack and the like (Jurkevitch and Davidov, 2007).

In a stricter sense, BALOs have newly been classified into three orders (Hahn et al., 2017), viz., order *Bacteriovoracales*, including families *Bacteriovoracaceae* (Davidov and Jurkevitch, 2004) (genera *Bacteriovorax* and *Peredibacter*), and *Halobacteriovoraceae* (Koval et al., 2015); order *Bdellovibrionales* (Brenner et al., 2005), only including genera *Bdellovibrio*, *Micarvibrio*, and *Vampirivibrio*, and other unclassified BALOs; and order *Oligoflexiales*, including family *Pseudobacteriovoracaceae*. All these three orders, viz., *Bdellovibrionales*, *Bacteriovoracales*, and *Oligoflexiales*, are placed under the class *Oligoflexia* (Nakai et al., 2014).

In a looser/broader sense, BALOs also include other Gram-negatives such as *Aristabacter necator*, *Cupriavidus necator*, *Ensifer adhaerens*, *Herpetosiphon* species of the *Chloroflexi* clade, genus *Lysobacter*, myxobacteria like *myxococcus*, a number of *Cytophaga* strains, and some strains of *Stenotrophomonas maltophilia* as well as Gram-positive bacteria such as *Agromyces ramosus*, *Streptoverticillium*, and even archae *Nanoarchaeum equitans* (Jurkevitch and Davidov, 2007).

Existing findings related to BALOs so far indicate that this group of bacteria can be used to control vibrios (e.g., Li et al., 2014; Guo et al., 2016), to alter or restore a microbial community structure (e.g., Li et al., 2014; Johnke et al., 2019), and to promote growth and survival of cultured organisms (Li et al., 2014; Guo et al., 2016). Therefore, in this study, our main aim is to apply BALOs on the rearing of second-generation shrimp PL after they have been subjected to salinity reduction treatment so as to reveal if BALOs could restore or rectify PL gut microbiota on the basis of high-throughput sequencing analysis. Results of this study could also explain, although indirectly, if salinity reduction treatment has any impact on PL gut microbiota.

## Materials and Methods

### Preparation of Host Strain

Gram-negative *Citrobacter amanolaticus* strain TC (GenBank accession number, MN956654) was isolated from salty water and used as a host for propagating BALOs. It was proven to be non-hemolytic (data not shown). Strain TC was grown in nutrient broth for 13–15 h at 30°C with shaking at 200 rev min^–1^ to reach the late exponential phase. Then it was harvested by centrifugation at 5000 rev min^–1^ for 10 min at 4°C and resuspended with sterile phosphate-buffered saline (PBS: 28 mmol L^–1^ NaH_2_PO_4_, 72 mmol L^–1^ Na_2_HPO_4_, pH 7.2) to the final concentration of 1 × 10^10^ CFU mL^–1^. It was stored at 4°C before use.

### Preparation of *Bdellovibrionales* Strain BDN-1F2 at Free Swimming Stage

BDN-1F2 was a mutant of wild-type BDN-1 after Co^60^ mutagenesis (data not shown). It lysed 90% of the 30 strains of bacteria tested (including nine strains of *Vibrio alginolyticus*, three strains of *V. parahaemolyticus*, four strains of *V. cholerae* non-0139/non-01, five strains of *Shewanella putrafaciens*, six strains of *Pseudomonas aeruginosa*, two strains of *Serratia ficaria*, and one strain of *Enterococcus agglomerans*) and 93.3% of 16 vibrio strains tested. These lysis rates are higher than its wild-type counterpart (86.7 and 87.5%, respectively) (Chen et al., 2019). Wild-type BDN-1 was identified as a strain of *Bdellovibrionales* (GenBank accession number, MK159102), closely related to *Bdellovibrionales* strain BDSH06 (GenBank accession number, EF011103) (Chen et al., 2019) (Supplementary Figure S1).

BDN-1F2 was kept as plaque on a 15‰ dilute nutrient broth (DNB: 0.8 g nutrient broth, 0.5 g casein hydrolyzate, 0.1 g yeast extract, 15 g salt, 1 L distilled water, pH 7.2) double-layer agar plate at 4°C before use. One single plaque was picked with a sterile inoculation loop from a freshly grown double-layer agar plate and inoculated into an Erlenmeyer flask that contained 50 mL DNB medium and 1 mL suspended host strain (Li et al., 2014) with vigorous shaking at 200 rev min^–1^ for 72 h at 28°C. After that, residue hosts were pelleted at 5000 rev min^–1^ for 20 min at 4°C, and the supernatant was filtered through 0.45-μm pore size membrane filter so as to rid the remaining hosts and debris. Filtrate was then centrifuged at 27,000 *g* and pellets were resuspended with sterile PBS to obtain final concentration 7.16 × 10^9^ plaque forming units (PFU) mL^–1^. It was kept at 4°C before use.

### Preparation of *Bacillus subtilis*

*Bacillus subtilis* GIM 1.136 was used as a potential probiont in this shrimp PL test. It was bought from Guangdong Institute of Microbiology and grown in nutrient broth for 13–15 h at 30°C with shaking at 200 rev min^–1^ to reach the late exponential phase. Then it was harvested by centrifugation at 5000 rev min^–1^ for 10 min at 4°C and the pellets were resuspended with sterile PBS (pH 7.2) to the final concentration 1 × 10^10^ CFU mL^–1^ and stored at 4°C before use.

### Preparation of Boiled Rearing Water and the Setup of Shrimp Postlarvae (PL) Tests

Postlarvae tests were conducted for 7 days at around 28°C in the laboratory.

Saltwater of 15‰ salinity was prepared by dissolving 15 g salt in 1 L of tap water and then boiled for 5 min and naturally cooled to room temperature. After that, it was aerated, and the dissolved oxygen (DO) concentration was brought back to 5 ppm or above with an air pump fitted with a 0.22-μm air-sterilization filter.

The purpose of using boiled water was to reduce possible microbe/vibrios contamination from the water and to reduce or even avoid interference of microbes/vibrios in water with PL gut microbiota as much as possible.

Nine plastic tanks with capacity of 6 L were disinfected with 0.1% KMnO_4_ and thoroughly rinsed with sterile saltwater. Then, 4 L of 15‰ boiled and aerated saltwater was poured into each tank. Aeration was done by keeping two air stones in each tank with a 0.22-μm membrane filter to filter out any possible bacterial contaminants in the aeration process.

Postlarval shrimp (*Litopenaeus vannamei*) of PL7-8 (body length: 0.7–0.8 cm on average) were gifted by a shrimp hatchery in Guangdong Province. They were second-generation PL and had been subjected to salinity reduction treatment before the salinity was brought back to 15‰ to meet our testing need. These PL7-8 were visually healthy with no apparent signs of diseases.

In total, every 70 PL were randomly assigned to each of the nine tanks. They were divided into three groups: CD, SD, and BD groups, each with triplicates. In the BD group, BDN-1F2 at the free-swimming stage was added to a final concentration of 1 × 10^4^ PFU mL^–1^. In the SD group, *Bacillus subtilis* GIM 1.136 was added to a final concentration of 1 × 10^6^ CFU mL^–1^. In the CD (control) group, nothing was added. BDN-1F2 and *Bacillus subtilis* GIM 1.136 were added to relevant groups once only at the start of the test.

PL were fed twice a day with 0.5 mg shrimp flakes (powder form) per 10 shrimp each time. The shrimp flakes contained 45% protein, 6% fat, 5% calcium, 1.2% phosphate, 1.4% lysine, 8% water, 16% ash, and 3% crude fiber.

No water was changed throughout the test period. Water temperature, DO, and pH were recorded daily. The number of dead shrimp was recorded, and the survival rates were calculated at the end of the test.

### Enumeration of BALOs, Total Cultivable Bacterial and Vibrios Counts

From each tank, water samples of 100 mL and 10 shrimp were randomly collected at days 0, 1, 3, 5, and 7 for counts of total cultivable bacteria, total vibrios, and BALOs. Water samples were first filtered with a 0.22-μM sterile membrane filter and then resuspended in 1 mL sterile PBS (pH 7.2), 10 × serially diluted for various counts.

In each group, all 10 shrimp were weighed together but rinsed separately: first with 75% ethanol and then with sterile distilled water to remove possible bacteria on body surfaces (Zheng et al., 2017). As PL7-8 shrimp were very tiny, it was practically impossible to dissect for the intestines (Zheng et al., 2017). Hence, the bodies of 10 PL in each replicate/sample were homogenized altogether using a sterile grinder with 1 ml sterile PBS (pH 7.2) and then divided into two parts: one for traditional bacteriological analysis as specified above and the other for high-throughput sequencing and stored at −20°C before use. Bacterial enumeration was performed in triplicate.

Total cultivable bacterial and vibrios counts were obtained by the spread plate method after incubation at 28°C for 24 h.

For total cultivable bacterial counts, marine 2216E (peptone 5 g, yeast extract 1 g, ferric phosphate 0.01 g, sodium chloride 15 g, agar 15 g, 1 L water, pH 7.6–7.8) was used. For total vibrios counts, thiosulfate citrate bile salts medium (TCBS) was used. A series of tenfold (10^0^, 10^–1^, 10^–2^, …) dilutions were made with sterile 15‰ saltwater and 0.1 mL of each dilution was spread onto appropriate culture medium (Guo et al., 2016); those plates having 30–300 colonies were counted and expressed as CFU mg^–1^ for PL shrimp samples and CFU mL^–1^ for water samples.

For BALOs counts, a double-layer plating technique was employed, viz., a 500-μL appropriately diluted sample and 500 μL of the host (strain TC) suspension (1 × 10^10^ CFU mL^–1^) were mixed with 3 mL of liquefied overlay agar (DNB medium containing 0.8% agar) that was kept in a thermostatic water bath at 50°C. The mixture was briefly vortexed to mix before being poured over the surface of a bottom-layer agar plate containing DNB medium with 1.5% agar in a Petri dish (90 mm in diameter). Plates were incubated at 28°C for 3–5 days until clear circular plaques appeared. Each plaque was counted as PFU, expressing as PFU mg^–1^ for PL samples or PFU mL^–1^ for water samples.

### Illumina High-Throughput Sequencing of Barcoded 16S rRNA Genes

PL gut microbiota was studied using Illumina high-throughput sequencing technology. MiSeq sequencing was performed after sampling, extraction of genome, and amplification of 16S rRNA gene sequences.

As gut microbiota in each individual farmed organism varies, a practice is to pool them together so as to avoid individual variations (Vadstein et al., 2013; Guo et al., 2017). Especially due to the salinity reduction treatment, the quantity of gut microbes in PL had been hugely reduced (as shown by the plate counts, 2–5 log CFU mg^–1^, Table 2), and initial PCR amplifications on individual replicate failed. To enable 16S rRNA gene high-throughput sequencing analysis to be done successfully as well as to possibly cover more minor taxa in gut microbiota, we, thus, pooled per group/time all three replicates together, viz., the aliquot-homogenized PL samples from replicate tanks in the same group (control or test) obtained on the same day were first mixed before running high-throughput sequencing as done by Li et al. (2014).

#### PL Shrimp Sample Preparation and DNA Extraction

PL samples of days 1 and 7 were used in this study as day 0 samples were lost by accident and no further analysis could be performed.

CD1, SD1, BD1 and CD7, SD7, and BD7 represented samples of control, *Bacillus subtilis*, and BDN-1F2 additions of days 1 and 7, correspondently.

Homogenized PL samples from three replicates were first pooled as mentioned above and then centrifuged at 10,000 rpm for 3 min, and the pellets were resuspended in double distilled water. Total bacterial genomic DNAs were extracted with E.Z.N.A^TM^ Mag-Bind Soil DNA Kit (Omega Bio-tek, Inc., GA, United States) according to the manufacturer’s instructions and stored at −20°C until use. The extracted genomic DNAs were checked for integrity by agarose gel electrophoresis.

#### PCR Amplification and MISeq Sequencing

Total bacterial genomic DNAs were used as template for the V3–V4 region amplification of the 16S rRNA gene, using a forward primer of 314F: 5′-CCCTACACGACGCTCTTCCGAT CTGCCTACGGGNGGCWGCAG-3′ and reverse primer 805R: 5′-GACTGGAGTTCCTTGGCACCCGAGAATTCCAGACTAC HVGGGTATCTAATCC-3′. The PCR amplification mixture contained 15 μL of 2 × Taq master Mix, 1 μL of barcoded-PCR primer F (10 μM), 1 μL of primer R (10 μM), and 10–20 ng of genomic DNA. Ultrapure sterile water was added to a final volume of 30 μL. PCR reaction was done with initial denaturation at 94°C for 3 min and then the first five cycles with denaturation at 94°C for 30 s, annealing at 45°C for 20 s and extension at 65°C for 30 s and then 20 cycles with denaturation at 94°C for 20 s, annealing at 55°C for 20 s, and extension at 72°C for 30 s. Final elongation was done at 72°C for 5 min. This was followed by a second amplification introduced with Illumina Bridge PCR Compatible Primers (Liu et al., 2017). The second PCR amplification mixture included 15 μL of 2 × Taq master Mix, 1 μL of primer F (10 μM), 1 μL of primer R (10 μM), 20 ng of PCR products (from the previous round). Ultrapure sterile water was added to a final volume of 30 μL. PCR reaction began with a 3-min predenaturation at 95°C; followed by five cycles of denaturation at 94°C for 20 s, annealing at 55°C for 20 s, extension at 72°C for 30 s, and a final elongation step at 72°C for 5 min.

PCR products were checked with agarose gel electrophoresis and purified using magnetic beads Agencourt AMPure XP (Sangon Biotech Co., Ltd., Shanghai, China). The Qubit2.0 DNA detection kit was used to quantify the recovered DNA. Each purified PCR product of 10 ng was pooled to a final sequencing concentration of 20 pmoL and then subjected to Illumina-based high-throughput sequencing (Sangon Biotech Co., Ltd., Shanghai, China).

#### Sequence Processing and Analysis

The raw Miseq files were first qualified by cutoff barcode, primers, and part of the low-quality reads (Window size 10 bp, Quality score < 20) using Cutadapt (version 1.2.1) and Prinseq (version 0.20.4). Usearch (version 5.2.236) was employed to wipe off the non-specific amplification sequence, and Uchime (version 4.20.40) was used for the identification and removal of Chimera. Clustering of operational taxonomic units (OTU) was performed using Usearch (version 5.2.236) based on a similarity cutoff of 97%. The phylogenetic affiliation of each 16S rRNA gene sequence was analyzed by RDP classifier^1^ against the SILVA database^2^ using confidence threshold of 70%.

Alpha and beta diversity metrics from the final OTU table without singletons were obtained using the QIIME 1.9.1. Taxonomic richness parameters, including ACE and Chao1 as well as community diversity parameters Shannon and Simpson and a sequencing depth index (Good’s coverage) that belong to Alpha diversity, were calculated using Mothur software (Schloss et al., 2009). Hierarchical clustering was conducted on the basis of beta diversity distance matrix and then the arboreal structure was constructed by an unweighted pair group method with arithmetic mean (UPGMA). Principal coordinate analyses (PCoA) based on OTU level were performed. Other relevant analyses were visualized with the R package software (version 3.2).

#### Functional Analysis

The Phylogenetic Investigation of Communities by Reconstruction of Unobserved States (PICRUSt) software package (version 1.0.0) was used to predict the metagenomic functional capacity using the final OTU table originated from 16S sequencing (Langille et al., 2013). PICRUSt uses an extended ancestral-state reconstruction algorithm to generate the composition of gene families for each metagenome (Huang B. H. et al., 2018). The Clusters of Orthologous Groups (COG) pathways obtained by PICRUSt was normalized and then produced a functional structure at a higher level among samples according to the functional prediction results, using a response ratio analysis at a 95% confidence interval. The relationships among functional capacities were analyzed by principal component analysis (PCA).

The raw 16S rDNA sequence data of high-throughput sequencing were deposited in the NCBI Sequence Read Archive (SRA) under the accession number PRJNA600113 (SRP241035 or SRR10861522-SRR10861527).

### Statistical Analysis

The data of general microbiology tests were expressed as mean ± standard deviation (*SD*) (*n* = 3), and Origin 8.0 (OriginLab, MA, United States) was used for statistical analysis and mapping. Statistically significant differences were determined by one-way analysis of variance (ANOVA), *p* < 0.05, using IBM SPSS statistics (V23, New York, United States). Pearson correlation coefficient analysis was also performed with IBM SPSS Statistics (V23, New York, United States). Other relevant statistical analyses were performed with R or PCRUSt software as mentioned above.

## Results

### Total Cultivable Bacteria Counts (TCBC) and Total Vibrios Counts (TVC) in PL Samples and Water

Throughout the 7-day test period, waters in all groups were relatively clear with a little feed residue at the bottom of the tanks. Water temperature was maintained at 28°C ± 0.5°C. DO was maintained at 5 ± 0.6 ppm, and pH was in the range of 7.5–7.9, in all groups.

The number of dead shrimp on average was 10 ± 2.83, 11 ± 2.16, and 6 ± 1.41 in the SD, CD, and BD groups, respectively, corresponding to the survival rates of 83.3% ± 4.7%, 81.7% ± 3.6%, and 90.0% ± 2.4% in the respective groups (Table 1). Although PL body length was not measured, PL body weight and percentage gains accumulated over the 7-day period were shown in Table 1. Clearly, PL in the BD group had the highest percentage accumulated weight gain (5.51% ± 0.34%) in all three groups (*p* < 0.05). Highest survival rate and percentage accumulated weight gain indicate that PL in the BD group grew best of all three groups.

**TABLE 1:** PL body weight and gains and survival rates in different groups over the 7-day test period (mg PL^–1^).

	Day 0	Day 1	Day 3	Day 5	Day 7	Weight gain (accumulated) (%)^1^	Survival rate (%)^2^
BD	5.83 ± 0.11^a^*	5.82 ± 0.14^*a*^	5.86 ± 0.22^a^	5.98 ± 0.62^a^	6.15 ± 0.52^a^	5.51 ± 0.34^a^	90.0 ± 2.4^a^
SD	5.84 ± 0.16^a^	5.84 ± 0.12^a^	5.85 ± 0.16^a^	5.91 ± 0.23^b^	5.94 ± 0.32^b^	1.70 ± 0.34^b^	83.3 ± 4.7^b^
CD	5.82 ± 0.17^a^	5.83 ± 0.15^a^	5.84 ± 0.18^a^	5.90 ± 0.31^b^	5.93 ± 0.24^b^	1.86 ± 0.17^b^	81.7 ± 3.6^b^

Data were given as means ± standard deviation (*n* = 3). ^1^Percentage accumulated weight gain in a group was calculated by first obtaining the sum of body weight at day 7 minus that at day 1, then divided by body weight at day 1, finally multiplied by 100%. ^2^Survival rate was calculated by the number of live PL at day 1 minus that at day 7, then multiplied by 100%. *Different letters in the same column indicate significant difference (p < 0.05), same letters indicate no difference (*p* > 0.05).

Although TVC was not detected on TCBS agar plates in shrimp and waters in all three groups throughout the 7-day test period, TCBC in PL and waters were given in Tables 2, 3, respectively.

**TABLE 2:** Total cultivable bacteria counts (TCBC) in PL shrimp (log CFU mg^–1^).

	Day 0	Day 1	Day 3	Day 5	Day 7
BD	5.26 ± 0.21^a^*	6.26 ± 0.13^a^	4.70 ± 0.11^b^	4.50 ± 0.09^b^	2.33 ± 0.15^c^
SD	5.45 ± 0.17^a^	6.97 ± 0.18^a^	5.12 ± 0.12^a^	5.77 ± 0.21^a^	5.61 ± 0.19^a^
CD	5.07 ± 0.19^a^	6.53 ± 0.14^a^	5.99 ± 0.19^a^	5.87 ± 0.15^a^	5.31 ± 0.18^b^

Data were given as means ± standard deviation (*n* = 3). No vibrios were detected on TCBS agar plates. *Different letters in the same column indicate significant difference (*p* < 0.05), same letters indicate no difference (*p* > 0.05).

**TABLE 3:** Total cultivable bacteria counts (TCBC) in waters (log CFU mL^–1^).

	Day 0	Day 1	Day 3	Day 5	Day 7
BD	5.58 ± 0.16^a^*	6.48 ± 0.18^a^	4.48 ± 0.15^b^	3.45 ± 0.11^b^	0.95 ± 0.12^c^
SD	5.02 ± 0.13^a^	6.04 ± 0.16^a^	5.15 ± 0.12^a^	5.02 ± 0.15^a^	4.61 ± 0.14^a^
CD	3.30 ± 0.18^b^	4.98 ± 0.11^b^	3.23 ± 0.17^c^	3.28 ± 0.13^b^	3.26 ± 0.16^b^

Data were given as means ± standard deviation (*n* = 3). No vibrios were detected on TCBS agar plates. *Different letters in the same column indicate significant difference (*p* < 0.05), same letters indicate no difference (*p* > 0.05).

Regarding TCBC in PL, their numbers were all over 5 log CFU mg^–1^ at day 0, up slightly at day 1, and then down (Table 2). Although the numbers in the SD and CD groups were maintained at 5 log CFU mg^–1^ level over the test period, it was gradually down to 2 log CFU mg^–1^ level in the BD group. All three groups showed significant differences at day 7 (*p* < 0.05).

With respect to TCBC in the water (Table 3), similar trends were noted as in PL (Table 2). The CD and SD groups maintained a relatively constant level throughout the 7-day period with their numbers at the levels of 3 log CFU mL^–1^ and 5 log CFU mL^–1^, respectively. In the BD group, TCBC was reduced from over 5 log CFU mL^–1^ level at day 0 to nearly just 1 log CFU mL^–1^ at day 7. Again, all three groups showed significant differences at day 7 (*p* < 0.05).

### Total BALOs Counts in PL and Waters

Double-layer plating with host strain TC was used for enumerating BALOs in PL and waters. They were only found in the BD group (Table 4) and not (zero counts) in the CD and SD groups.

**TABLE 4:** Total BALOs counts of BD group in water (log PFU mL^–1^) and PL shrimp (log PFU mg^–1^).

	Day 0	Day 1	Day 3	Day 5	Day 7
Water	4.76 ± 0.11	4.57 ± 0.14	3.51 ± 0.20	3.04 ± 0.10	2.83 ± 0.16
PL	N*	2.91 ± 0.15	2.24 ± 0.13	1.03 ± 0.17	1.79 ± 0.18

Double-layer plating with host strain TC was used for enumerating BALOs in PL and waters. BALOs were only detected in BD group, and not (zero counts) in CD and SD groups. *N, not assayed, as BDN-1F2 was just added to the waters and not so likely to reach PL guts instantly.

In the water, BALOs were over 4 log PFU mL^–1^ at days 0 and 1 and then gradually down to 2 log PFU mL^–1^ at the end of the test. In PL, their numbers were around 2 log PFU mg^–1^ at day 1 and then gradually down to 1 log PFU mg^–1^ in the end.

Pearson correlation analysis was performed so as to identify any potential correlations among bacterial (BALOs, TCBC) and PL growth parameters (Supplementary Table S1). Results showed that BALOs (BDN-1F2) displayed a statistically significant correlation with TCBC in water only (*p* < 0.05).

### Illumina High-Throughput Sequencing Analysis

#### Overview of 16S RNA Gene Sequencing

In total, 361,502 raw reads were yielded from six samples belonging to the CD, SD, and BD groups. After quality control, including cutoff barcode, excluding primers and part of the low-quality reads as well as chimera and non-specific amplification sequences, on average, 53,912 sequence (seq) numbers were obtained, ranging from 42,258 seq number to 69,351 seq number. The sequence number, coverage, and statistical estimates of richness and diversity indices from each group were given in Table 5.

**TABLE 5:** Diversity and richness indices relative to each sample.

Sample ID	Seq num	OTU Num^1^	Shannon Index^2^	ACE index^3^	Chao1 index^3^	Coverage^4^	Simpson index^2^
BD1	42258	526	0.93	1719.43	1018.77	0.99	0.66
BD7	47169	3395	7.14	3402.66	3396.20	1.00	0.01
SD1	69351	283	0.98	496.37	426.57	1.00	0.66
SD7	58766	640	0.93	1034.53	935.02	0.99	0.70
CD1	49884	646	2.94	819.58	736.27	1.00	0.11
CD7	56046	629	0.94	997.27	906.07	0.99	0.73

*^1^OTUs were defined at the 97% sequence similarity level. ^2^Shannon and Simpson index were community diversity parameters. A higher number of Shannon index indicates a higher diversity, whereas a higher number of Simpson indicates a lower diversity. ^3^ACE index and Chao1 index were community richness parameters. A higher number indicates more richness. ^4^Coverage was the sequencing depth index, re*fl*ecting if the sequencing result represents the real situation of a sample. A higher number indicates lower probability that the sequence is not detected in the sample. Coverage of 1.00 means all the possible species in a sample has been covered in the sequencing.*

Sequences at the 97% similarity were clustered as an OTU. Observed OTU richness and numbers of unique OTUs revealed that a highest OTU number of 3395 was with BD7 and a lowest of 283 with SD1 with the rest in between (526–649) (Table 5).

As coverage was the sequencing depth index, a value of 1.00 or near 1.00 indicates all or nearly all the species in a sample have been represented in the sequencing as is the case with this study (Table 5). This means that the sequencing results represent the real situation of each sample.

#### Alpha and Beta Diversity of Bacterial Communities

Alpha diversity of a bacterial community includes Shannon and Simpson indices, which reflect the extents of community diversities. A higher number of on the Shannon index indicates a higher diversity, whereas a higher number on Simpson indicates a lower diversity.

Alpha diversity of a bacterial community also includes ACE index and Chao1 index, which reflect the extents of community richness. A higher number indicates more richness.

The diversity and richness indices of each sample are also given in Table 5.

Clearly, with the addition of BDN-1F2, PL microbial diversities in the BD group were improved exceedingly with Shannon index rising from 0.93 at day 1 to 7.14 at day 7, and the Simpson index dropped from 0.66 to 0.01.

PL microbial diversities in the SD group were initially nearly the same as that in the BD group (Shannon index 0.98, Simpson index 0.66 at day 1), and they were still no better after the addition of *Bacillus subtilis* with Shannon and Simpson indices at 0.93 and 0.70 at day 7, respectively.

In control (CD), where no probiotics were added, PL microbial diversities became worse over the 7-day test period with Shannon index down from 2.94 to 0.94 and Simpson index up from 0.11 to 0.73 at day 1 and 7, respectively.

Regarding the richness of PL microbial communities, ACE and Chao1 indices both showed that the BD group had the highest values of all, nearly fourfold as much as the other groups at day 7 (Table 5), indicating that PL microbiota in the BD group were much more robust than those in the SD and CD groups at the end of the test.

Beta diversity analysis was to show the similarities and differences of microbial communities of different samples. Both the hierarchical clustering tree (not shown) and PCoA analyses (not shown) on all groups revealed that BD7 was far away from the rest, manifesting that BDN-1F2 addition had impacted PL microbiota in the BD group significantly (*p* < 0.05) while *Bacillus subtilis* addition in the SD group did not.

#### Taxonomic Compositions and Changes of PL Microbial Communities

**FIGURE 1:**
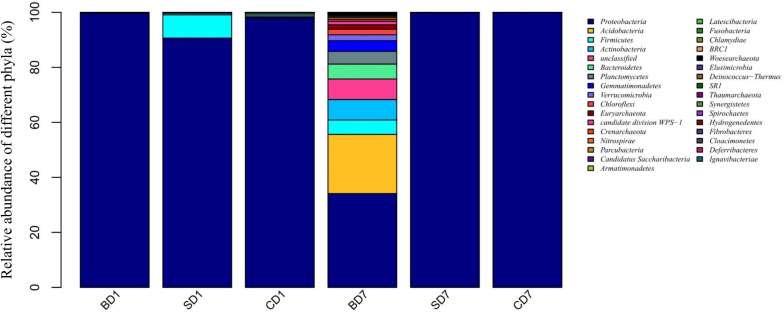
Relative abundance of different bacterial phyla within PL microbiota in BD, SD, and control (CD) groups. This distribution bar plot was drawn with R software. BD1, SD1, and CD1 represent PL samples at day 1 of the test, and BD7, SD7, and CD7 represent PL samples at day 7.

As revealed by high-throughput sequencing, there were 33 phyla detected in the PL microbiota in total, but only 11 phyla were shown to have an abundance of ≥1% (Figure 1 and Table 6). At day 1, *Proteobacteria* was the only dominant phylum in all groups with an abundance of 99.7, 90.45, and 97.77% in BD1, SD1, and CD1, respectively.

**TABLE 6:** Relative abundance of dominant orders in each sample^1^.

Phylum or Class/Order	Relative abundance (%)					
	BD1	SD1	CD1	BD7	SD7	CD7
***Gammaproteobacteria***						
*Pseudomonadales*	87.63	84.97	59.95	2.04	99.73	99.88
*Oceanospirillales*	0.01	0.07	19.75	0.04	0	0
*Xanthomonadales*	11.71	0	0.02	2.02	0	0
*Enterobacteriales*	0.02	0.32	0.75	3.56	0.04	0.01
*Legionellales*	0	0	0	0.32	0	0
*Chromatiales*	0.06	0.24	2.78	0.09	0	0
*Alteromonadales*	0	0.01	0.03	0.37	0	0
Total	99.43	85.61	83.28	8.44	99.77	99.89
***Alphaproteobacteria***						
*Rhodobacterales*	0.21	4.59	13.78	0.4	0.06	0.04
*Sphingomonadales*	0	0.07	0.1	6.56	0.01	0
*Rhizobiales*	0.05	0.08	0.13	4.16	0.01	0
*Rhodospirillales*	0	0	0	1.42	0	0
*Caulobacterales*	0	0.06	0.01	0.4	0	0
Total	0.26	4.8	14.02	12.94	0.08	0.04
***Betaproteobacteria***						
*Burkholderiales*	0.01	0.11	0.47	3.64	0.01	0.01
*Rhodocyclales*	0	0	0.03	1.68	0	0
Total	0.01	0.11	0.5	5.32	0.01	0.01
***Deltaproteobacteria***						
*Myxococcales*	0	0	0.01	2.49	0	0
*Bdellovibrionales*	0	0	0.01	0.3	0	0
*Desulfuromonadales*	0.03	0	0	0.36	0	0
Total	0.03	0	0.02	3.15	0	0
**Unclassified**	0.05	0.11	0.44	38.7	0.05	0.01
***Acidobacteria***						
*Acidobacteria_*Gp6	0	0.04	0.13	10	0.01	0
*Acidobacteria_*Gp4	0	0.01	0.06	6.25	0.01	0
*Acidobacteria_*Gp7	0	0	0.03	1.3	0	0
*Acidobacteria_*Gp16	0	0	0.03	1.23	0	0
Total	0	0.05	0.25	18.78	0.02	0
***Firmicutes***						
*Bacillales*	0.12	8.35	0.04	1.09	0	0.01
*Lactobacillales*	0	0.06	0.05	2.03	0.01	0
*Selenomonadales*	0	0	0.02	0.34	0	0
*Clostridiales*	0	0.02	0.04	1.46	0	0
Total	0.12	8.43	0.15	4.92	0.01	0.01
***Actinobacteria***						
*Actinomycetales*	0	0.56	0.56	3.4	0	0
*Acidimicrobiales*	0	0.01	0.02	2.25	0	0
*Gaiellales*	0	0	0.01	0.99	0	0
Total	0	0.57	0.59	6.64	0	0
***Gemmatimonadetes***						
*Gemmatimonadales*	0	0.01	0.02	4.03	0	0
***Planctomycetes***						
*Planctomycetales*	0	0.02	0.09	3.92	0	0.01
***Bacteroidetes***						
*Sphingobacteriales*	0	0.01	0.1	2.58	0	0
*Cytophagales*	0.01	0.01	0.01	1.28	0	0
*Bacteroidales*	0	0	0.05	0.85	0	0
*Flavobacteriales*	0.05	0.18	0.41	0.32	0	0
Total	0.06	0.2	0.57	5.03	0	0
***Chloroflexi***						
*Anaerolineales*	0	0.01	0.01	1.42	0	0
**Candidate division WPS-1**						
Candidate division WPS-1	0	0.01	0.01	1.21	0	0
***Euryarchaeota***						
*Methanosarcinales*	0.01	0.01	0.05	0.97	0.01	0
*Methanomassiliicoccales*	0	0.01	0.02	0.47	0	0
Total	0.01	0.02	0.07	1.44	0.01	0
***Thaumarchaeota***						
*Nitrospirales*	0	0	0.01	0.68	0	0
***Verrucomicrobia***						
*Opitutales*	0	0	0	0.38	0	0
*Verrucomicrobiales*	0	0	0.01	0.42	0	0
Total	0	0	0.01	0.8	0	0

*^1^A relative abundance of 0.3% or above in a microbiota as revealed by high-throughput sequencing was considered as a dominant/major order. *Proteobacteria*, including *Alphaproteobacteria*, *Betaproteobacteria*, *Gammaproteobacteria*, *Deltaproteobacteria*, consisted of 99.75, 90.58, 97.99, 34.13, 99.89, 99.97% of PL microbiota in BD1, SD1, CD1, BD7, SD7 and CD7 respectively.*

At the end of the 7-day test period, although *Proteobacteria* was still the only dominant phylum in the SD and CD groups with an abundance of 99.86 and 99.94% in SD7 and CD7 correspondingly, more diverse dominant phyla were detected in the BD group, including *Proteobacteria* (34.13%), *Acidobacteria* (21.51%), unclassified (7.46%), *Actinobacteria* (7.43%), *Bacteroidetes* (5.43%), *Firmicutes* (5.24%), *Planctomycetes* (4.6%), *Verrucomicrobia* (2.13%), *Chloroflexi* (2.03%), and *Euryarchaeota* (1.66%).

At the order level (Supplementary Figure S2 and Table 6), when we considered a relative abundance of 0.3% or above in microbiota as a major order, there were 42 orders in total. *Pseudomonadales* was the predominant order in all groups at day 1 with its relative abundance at 87.63, 84.97, and 59.95% in BD1, SD1, and CD1, respectively. At day 7, although its predominance was further strengthened in the SD (99.73%) and CD groups (99.88%), its relative abundance was sharply reduced to merely 2.04% in the BD group. An unclassified grouping replaced *Pseudomonadales* as the dominant order with relative abundance reaching 38.70% in the BD group (Table 6).

**FIGURE 2:**
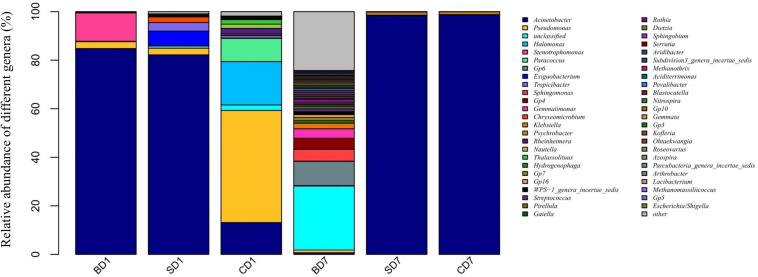
Relative abundance of different bacterial genera within PL microbiota in BD, SD, and control (CD) groups. This distribution bar plot was drawn with R software. BD1, SD1, and CD1 represent PL samples at day 1 of the test, and BD7, SD7, and CD7 represent PL samples at day 7.

At the genus level (Figure 2 and Table 7), when we consider an abundance of ≥1% in a microbial community as a dominant genus as for phylum, the number and abundance of dominant genera in different groups varied widely.

**TABLE 7:** Changes of relative abundance of various major bacterial genera in each sample over the 7-day test period^1^.

Genus	BD1	BD7	Changes (%)^2,3^	SD1	SD7	Changes (%)	CD1	CD7	Changes (%)
*Acinetobacter*	84.81	0.68	–84.13	82.19	98.41	+ 16.22	13.12	98.72	+ 85.6
*Chryseomicrobium*	0.12	0.04	–0.08	2.34	0	–2.34	0	0	N
*Exiguobacterium*	0	0.05	+ 0.05	5.99	0	–5.99	0	0	N
*Gemmatimonas*	0	3.9	+ 3.9	0.01	0	–0.01	0.02	0	–0.02
Gp4	0	4.58	+ 4.58	0	0.01	+ 0.01	0.05	0	–0.05
Gp6	0	10	+ 10	0.04	0.01	–0.03	0.13	0	–0.13
Gp7	0	1.3	+ 1.3	0	0	N	0.03	0	–0.03
Gp16	0	1.23	+ 1.23	0	0	N	0.03	0	–0.03
*Halomonas*	0.01	0.01	Invariable	0.07	0	–0.07	17.89	0	–17.89
*Hydrogenophaga*	0	0.96	+ 0.96	0.07	0	–0.07	0.26	0	–0.26
*Klebsiella*	0.02	2.28	+ 2.26	0.29	0.01	–0.28	0.54	0.01	–0.54
*Nautella*	0.1	0	–0.1	0.12	0	–0.12	1.77	0.01	–1.76
*Paracoccus*	0	0.12	+ 0.12	0.09	0	–0.09	9.53	0	–9.53
*Pseudomonas*	2.8	1.11	–1.69	2.75	0.01	–2.74	46.16	0.01	–46.15
*Psychrobacter*	0	0.04	+ 0.04	0	1.31	+ 1.31	0.01	1.15	+ 1.14
*Sphingomonas*	0	4.83	+ 4.83	0.02	0.01	–0.01	0.04	0	–0.04
*Rheinheimera*	0	0	N	0.13	0	–0.13	2.27	0	–2.27
*Stenotrophomonas*	11.71	0.07	–11.64	0	0	N	0	0	N
*Streptococcus*	0	1.13	+ 1.13	0.03	0	–0.03	0.01	0	–0.01
*Thalassolituus*	0	0	N	0	0	N	1.84	0	–1.84
*Tropicibacter*	0.03	0.03	Invariable	3.48	0.05	–3.43	1.04	0.01	–1.03
Unclassified	0.2	26.35	+ 26.15	0.85	0.05	–0.8	2.28	0.03	–2.25
WPS-1-genera-incertae-sedis	0	1.21	+ 1.21	0.01	0	–0.01	0.01	0	–0.01

*^1^A dominant genus was defined as its relative abundance was ≥1% of microbiota composition. ^2^Percentage changes (%) was calculated by first obtaining the sum of the relative abundance at day 7 minus that at day 1, then divided by the relative abundance at day 7. The difference was finally multiplied by 100%. ^3^ N means not detected, so not applicable.*

At day 1, five dominant genera were found in the SD group (*Acinetobacter*, 82.19%; *Exiguobacterium*, 5.99%; *Tropicibacter*, 3.48%; *Pseudomonas*, 2.75%; *Chryseomicrobium*, 2.34%), nine in the CD group (*Pseudomonas*, 46.16%; *Halomonas*, 17.89%; *Acinetobacter*, 13.12%; *Paracoccus*, 9.53%; unclassified, 2.28%; *Rheinheimera*, 2.27%; *Thalassolituus*, 1.84%; *Nautella*, 1.77%; and *Tropicibacter*, 1.04%), and only three in BD group (*Acinetobacter*, 84.81%; *Stenotrophomonas*, 11.71%; *Pseudomonas*, 2.8%).

At day 7, the number of dominant genera was reduced to only two in both the CD (*Acinetobacter*, 98.72%; *Psychrobacter*, 1.15%) and SD group (*Acinetobacter*, 98.41%; *Psychrobacter*, 1.31%), whereas its number was expanded to 11 in the BD group (unclassified, 26.35%; Gp6, 10%; *Sphingomonas*, 4.83%; Gp4, 4.58%; *Gemmatimonas*, 3.90%; *Klebsiella*, 2.28%; Gp7, 1.30%; Gp16, 1.23%; WPS-1_genera_incertae_sedis, 1.21%; *Streptococcus*, 1.13%; and *Pseudomonas*, 1.11%).

With regard to the extent of changes in different genera in three groups over the 7-day test period (Table 7), *Acinetobacter* came first as it had an 84.13% reduction in the BD group with an 85.6% increment in the CD group at the opposite end. *Pseudomonas* came second as it displayed a reduction of 46.15% in the CD group and 1–2% in both the BD and SD groups.

In the BD group, two extra genera showing huge increments in abundance were the unclassified genus (26.15%) and *Acidobacteria*-Gp6 (10%).

As a special group of bacteria, none of the predatory bacteria were detected in PL in the SD and BD groups (if *Stenotrophomonas* was not counted here) at day 1. On contrast, three genera existed in PL of the CD group, viz., *Ensifer*, *Nitrospira*, and *Peredibacter*, all at an abundance of 0.01% in CD1 (Table 8).

**TABLE 8:** Relative abundance of genera being recognized in the BALOs grouping.

Genus	Relative abundance (%)						References
	BD1	SD1	CD1	BD7	SD7	CD7	
*Agromyces*	0	0	0	0.01	0	0	Jurkevitch and Davidov, 2007
*Bdellovibrio*	0	0	0	0.1	0	0	Stolp and Starr, 1963
*Cupriavidus*	0	0	0	0.04	0	0	Jurkevitch and Davidov, 2007
*Ensifer*	0	0	0.01	0.09	0	0	Jurkevitch and Davidov, 2007
*Halobacteriovorax*	0	0	0	0.01	0	0	Koval et al., 2015
*Herpetosiphon*	0	0	0	0.01	0	0	Jurkevitch and Davidov, 2007
*Myxococcus*	0	0	0	0.09	0	0	Jurkevitch and Davidov, 2007
*Nitrospira*	0	0	0.01	0.65	0	0	Dolinsek et al., 2013
*Peredibacter*	0	0	0.01	0.1	0	0	Jurkevitch and Davidov, 2007
*Stenotrophomonas*^1^	11.71	0	0	0.07	0	0	Jurkevitch and Davidov, 2007
*Vampirovibrio*	0	0	0	0.08	0	0	Jurkevitch and Davidov, 2007

*^1^Some strains of *Stenotrophomonas* are considered as BALOs (Jurkevitch and Davidov, 2007).*

At the end of the test, BALOs compositions and abundances have changed with none in the SD and CD groups and 11 genera in the BD group, viz., *Agromyces*, *Bdellovibrio*, *Cupriavidus*, *Ensifer*, *Halobacteriovorax*, *Herpetosiphon*, *Lysobacter*, *Myxococcus*, *Nitrospira*, *Peredibacter*, *Vampirovibrio* (Table 8). This means that the previously existing BALOs in control were gone or below detection levels while, in the BD group, their richness and diversities have been enhanced after the addition of BDN-1F2.

As another special group of procaryotes, *Archaea* was also picked up in this high-throughput sequencing (Table 9) and had a percentage of 0.1% (CD group) or less (0.03% in the SD group and 0.02% in the BD group) in relative abundance at day 1. However, at day 7, its share had risen to 2.0% in the BD group or been reduced to 0.02% in the SD group or 0.05% in the CD group.

**TABLE 9:** Relative abundance of *Archaea* in PL gut microbiota and their compositions.

Percentage of *Archaea* in PL gut microbiota (%)^1^			BD1	SD1	CD1	BD7	SD7	CD7
	
			0.02	0.03	0.1	2.0	0.02	0.05

Within *Archaea*			Percentage of different archaeal taxonomic groupings in PL guts within each group (%)					
Phyla	Class	Order						
*Euryarchaeota*	*Methanomicrobia*	*Methanosarcinales*	50	30	42	39	60	33
		*Methanomicrobiales*	0	9	8	4	20	33
	*Methanobacteria*	*Methanobacteriales*	10	17	15	5.3	0	0
	*Halobacteria*	*Halobacteriales*	0	0	0	0.9	0	0
	*Thermoplasmata*	*Methanomassiliicoccales*	0	26	15	19	10	0
	Unclassified	Unclassified	0	0	0	1.2	0	0
*Crenarchaeota*	*Thermoprotei*	*Desulfurococcales*	0	0	3	2	0	0
		Unclassified	40	17	17	27	10	33
*Thaumarchaeota*	*Nitrososphaeria*	*Nitrososphaerales*	0	0	0	1	0	0
		*Nitrosopumilales*	0	0	0	0.2	0	0
*Woesearchaeota*	–^2^	Incertae sedis AR16^2^	0	0	0	2.0	0	0
	–^2^	Incertae sedis AR20^2^	0	0	0	0.3	0	0
	Unclassified	Unclassified	0	0	0	0.3	0	0
Unclassified	Unclassified	Unclassified	0	0	3	0	0	0

*^1^Percentage (%) of *Archaea* in gut microbiota in each sample was revealed by the 16S rDNA high-throughput sequencing. ^2^Means that further classification is undetermined at present.*

With respect to archaeal taxonomic compositions in PL microbiota (Table 9), three phyla (*Crenarchaeota*, *Euryarchaeota*, and Unclassified) were present in the CD group and two (*Crenarchaeota* and *Euryarchaeota*) in the SD and BD groups at day 1. At day 7, both the SD and CD groups had only two phyla (*Crenarchaeota* and *Euryarchaeota*), while the BD group had expanded to encompass five phyla, viz., *Euryarchaeota*, *Thaumarchaeota*, *Thermoprotei*, *Woesearchaeota*, and unclassified.

#### Bacterial Interactions at Various Taxonomic Levels

Correlations between bacteria have been employed to reveal their possible positive or negative interactions in a microbial community. In this study, the first 100 OTUs in relative abundance were examined for their potentially positive or negative interactions with statistical significance (*p* < 0.05), using the SparCC approach, which has been shown to be “able to accurately recapitulate much of the known correlation structure from relative abundance data” (Friedman and Alm, 2012; Carr et al., 2019). Their network graphs were drawn with the igraph package in R software or with the Kamada–Kawai algorithm. Positive interactions indicate their interrelationships are synergistic, and negative interactions mean antagonistic.

**FIGURE 3:**
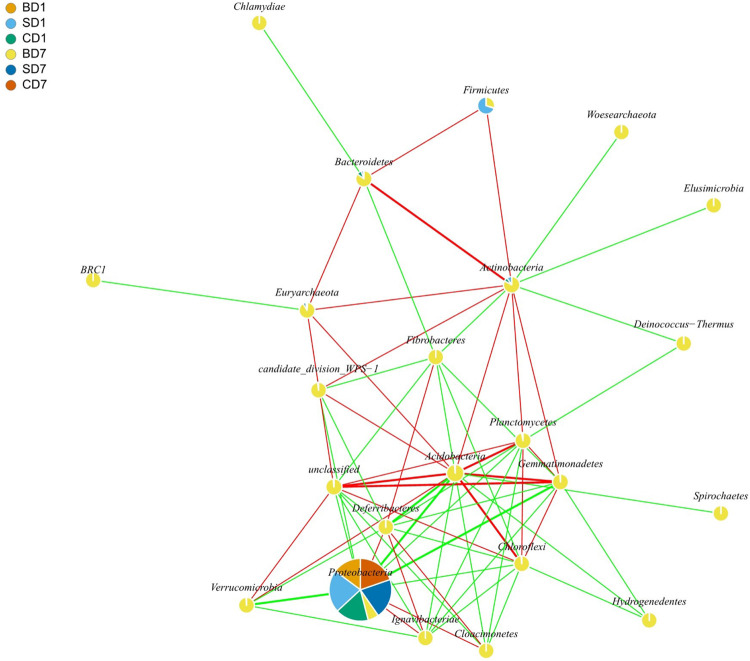
Network interaction graph for microbial communities of BD, SD, and CD groups at the phylum level, using SparCC approach (Friedman and Alm, 2012). Each node represents a bacterial phylum, each color in a node represents a relative abundance of a sample. When a *p*-value is between 0.01 and 0.05, the correlation is significant, and a dashed line is used; when a value is *p* < 0.01, the correlation is highly significant, and a solid line is used. When the correlation is positive, a red color line is used; when a correlation is negative, a green color line is used. When a correlation coefficient is >0.8, a thick line is used; when a correlation coefficient is <0.8, a thin line is used. In drawing this graph (with igraph package in R software), only those data that showed correlation coefficients higher than 0.6 in absolute value and were statistically significant (*p* < 0.05) were used. For the sake of simplicity as well as for showing more data, interactions at the phylum level are shown here rather than at the order level, which was given in the form of Supplementary Table S2. BD1, SD1, and CD1 represent PL samples at day 1 of the test, and BD7, SD7, and CD7 represent PL samples at day 7.

Results of interaction analyses at the phylum level are shown in Figure 3. For example, statistically significant and highly strong positive correlations (coefficient ≥ 0.8) were found in pairs such as *Acidobacteria* versus *Chloroflexi*, *Bacteroidetes* versus *Actinobacteria*, and *Planctomycetes* versus *Acidobacteria*, implying that they have very strong synergistic interrelationships (*p* < 0.05). Statistically significant and highly strong negative correlations (coefficient ≤ −0.8) were found in pairs such as *Acidobacteria* versus *Proteobacteria*, *Gemmatimonadetes* versus *Proteobacteria*, and *Verrucomicrobia* versus *Proteobacteria*. These results mean that *Acidobacteria*, *Gemmatimonadetes*, and *Verrucomicrobia* can strongly inhibit the growth of *Proteobacteria* (*p* < 0.05).

Results of interaction analyses at the order level are shown in Supplementary Table S2. Statistically significant positive correlations (coefficient ≥ 0.6) were found in PL microbiota, in pairs such as *Actinomycetales* versus *Bacteroidales*, *Bdellovibrionales*, *Bacillales*, *Burkholderiales*, or *Xanthomonadales* (*p* < 0.05); *Aeromonadales* versus *Methanomicrobiales*, *Halobacteriales*, *Rhodobacterales*, or *Pseudomonadales* (*p* < 0.05); *Bdellovibrionales* versus *Bacillales*, *Burkholderiales*, *Nitrospirales*, or *Xanthomonadales* (*p* < 0.05); and *Pseudomonadales* versus *Vibrionales* (*p* < 0.05). Statistically significant negative correlations (coefficient ≤ −0.6) were found in pairs such as *Aeromonadales* versus *Lactobacillales*, *Rhizobiales*, *Myxococcales*, *Actinomycetales*, *Bacteroidales*, *Bdellovibrionales*, *Bacillales*, or *Xanthomonadales* (*p* < 0.05); *Pseudomonadales* versus *Rhizobiales*, *Myxococcales*, *Actinomycetales*, *Bacteroidales*, *Bdellovibrionales*, *Bacillales*, *Burkholderiales*, *Nitrospirales*, or *Xanthomonadales* (*p* < 0.05); and *Vibrionales* versus *Rhizobiales*, *Myxococcales*, *Actinomycetales*, *Bacteroidales*, *Bdellovibrionales*, *Bacillales*, *Burkholderiales*, *Nitrospirales*, or *Xanthomonadales* (*p* < 0.05).

At the genus level (Supplementary Figure S3 and Supplementary Table S3), correlation analysis also revealed statistically significant positive (coefficient ≥ 0.6) pairs such as *Actinomyces* versus *Flavisolibacter* (*p* < 0.05); *Bacillus* versus *Lactobacillus*, *Pseudomonas*, or *Sphingomonas* (*p* < 0.05); and *Lactobacillus* versus *Pseudomonas* or *Acinetobacter* (*p* < 0.05) and negative (coefficient ≤ −0.6) pairs such as *Actinomyces* versus *Parasegetibacter*, *Niastella*, *Nitrospira*, *Lacibacterium*, or *Mycobacterium* (*p* < 0.05); *Bacillus* versus *Actinomyces* or *Hydrogenophaga* (*p* < 0.05); and *Lactobacillus* versus *Nitrospira*, *Pirellula*, *Actinomyces*, *Hydrogenophaga*, or *Streptococcus* (*p* < 0.05).

From the correlation analyses, it is quite clear that bacteria from orders such as *Actinomycetales*, *Bacillales*, *Bacteroidales*, *Bdellovibrionales*, *Lactobacillales*, *Myxococcales*, and *Rhizobiales* are antagonistic against those well-recognized opportunistic pathogens in *Aeromonadales* and *Vibrionales*, indicating that the current practice of selecting/applying probiotic strains from these orders for use in aquaculture has very sound and solid scientific foundations. Furthermore, addition of potentially beneficial bacteria such as BALOs could also promote other potentially beneficial microbe growth (positive correlation such as *Bdellovibrionales* vs. *Bacillales*, *Burkholderiales*, *Nitrospirales*, or *Xanthomonadales*) although limited to certain extents [as revealed by the negative correlations at the genus level, e.g., *Bacillus* vs. *Actinomyces* or *Hydrogenophaga* (*p* < 0.05) and *Lactobacillus* vs. *Nitrospira*, *Pirellula*, *Actinomyces*, *Hydrogenophaga*, or *Streptococcus* (*p* < 0.05)]. This fits in well with the concept of a balanced ecosystem that excels at its functions as overgrown microbes, even probiotics, could disrupt an ecosystem’s balance and, thus, proper functions.

#### Functional Prediction of PL Microbiota

**FIGURE 4:**
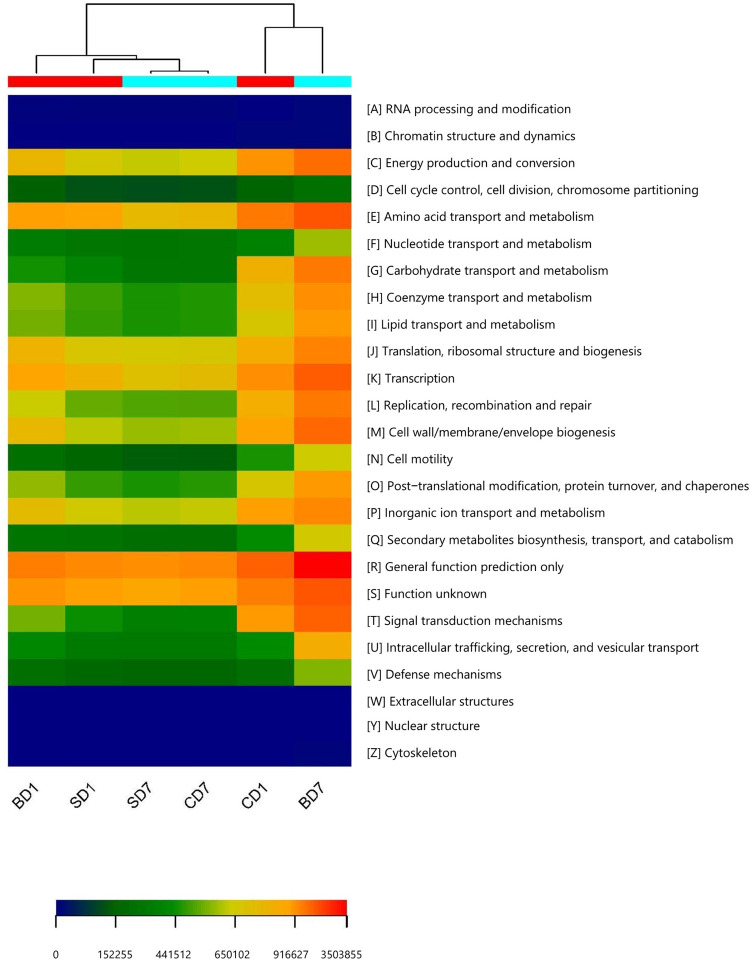
Heat map of predicted functions based on COG analysis with PICRUSt. The predictions have 95% probabilities in truly reflecting the real functions of a gut microbiota (*p* < 0.05). The heat map was drawn with the gplots package in R software. Different colors represent the extent of strength/richness of a function with a darker red color representing stronger strength (higher richness) and darker blue representing lower richness. BD1, SD1, and CD1 represent PL samples at day 1 of the test, and BD7, SD7, and CD7 represent PL samples at day 7.

As functional predictions in the PICRUSt on the basis of KEGG were found to be more related to human, we turned to COG for the functional predictions on PL microbiota in groups of CD (control), SD (addition of *Bacillus subtilis* GIM 1.136), and BD (addition of BDN-1F2). The detailed results are given in Figure 4 and Table 10.

**TABLE 10:** Predicated functions of PL microbiota and their relative percentages (strength) in CD, SD and BD groups on the basis of COG analysis in the PICRUSt^1^.

Predicted functions	Sequence hits in CD1^2^	SD1 vs. CD1 (%)^3^	BD1 vs. CD1 (%)	Sequence hits in CD7	SD7 vs. CD7 (%)	BD7 vs. CD7 (%)
RNA processing and modification	12101	198.81**	159.68**	21626	98.62**	138.43**
[B] Chromatin structure and dynamics	32062	48.63**	33.28**	11077	104.62	315.35**
[C] Energy production and conversion	2795119	86.09**	60.44**	1829647	102.61	231.17*
[D] Cell cycle control, cell division, chromosome partitioning	418443	108.03**	76.88**	365147	101.35**	164.07**
[E] Amino acid transport and metabolism	4049675	78.49**	50.72**	2236313	102.74	225.01**
[F] Nucleotide transport and metabolism	942066	111.64**	75.23**	821300	102.60	172.87**
[G] Carbohydrate transport and metabolism	2090219	63.72**	46.11**	832215	103.18*	461.05**
[H] Coenzyme transport and metabolism	1883053	92.26**	64.38**	1332183	102.66	221.47**
[I] Lipid transport and metabolism	1737340	99.31**	68.16**	1322661	103.05*	186.28**
[J] Translation, ribosomal structure and biogenesis	2137244	113.36**	80.99**	1942789	102.69	173.76**
[K] Transcription	3160908	91.81**	61.26**	2135100	102.51**	226.71**
[L] Replication, recombination and repair	2141633	87.99**	64.14**	1470392	104.14**	259.25**
[M] Cell wall/membrane/envelope biogenesis	2280367	95.82**	73.68**	1696726	102.86	257.66**
[N] Cell motility	1155279	53.83**	47.45**	411624	101.51**	376.62**
[O] Post-translational modification, protein turnover, and chaperones	1749521	97.69**	71.49**	1355552	101.64**	183.21**
[P] Inorganic ion transport and metabolism	2447045	95.09**	66.12**	1795318	103.98**	168.70**
[Q] Secondary metabolites biosynthesis, transport, and catabolism	1102709	84.22**	55.62**	669183	103.00	236.36**
[R] General function prediction only	4745894	99.41**	68.19**	3581794	102.63*	230.71**
[S] Function unknown	3621424	93.51**	66.11**	2570393	102.89	193.14**
[T] Signal transduction mechanisms	2720944	57.67**	43.65**	1022903	102.47*	437.66**
[U] Intracellular trafficking, secretion, and vesicular transport	1101219	101.28**	80.43**	901110	102.07**	226.26**
[V] Defense mechanisms	603283	105.40**	83.14**	498363	103.51**	272.40**
[W] Extracellular structures	1850	2.27**	0.16**	1	100.00	30500.00**
[Y] Nuclear structure	0	0.00	0.00	0	0.00	0.00
[Z] Cytoskeleton	1047	25.41**	2.77**	7	542.86**	235685.71**

^1^In the PICRUSTs, KEGG was not appropriate as it gave out analysis more related to humans, while COG analysis we found was more suitable for this work. ^2^Sequence hits (numbers) in CD1 and CD7 were directly cited from the outputs of COG analysis and treated as 100% percentiles in each predicted function at day 1 and 7, respectively. ^3^Percentages in each predicated function in SD and BD groups at day 1 or 7 were calculated as follows when compared to controls (CD1, CD7): the hit number in SD or BD group minus the hit number in CD on the same day, then the sum was divided by the hit number in CD of the same day and multiplied by 100 (%). The percentages thus obtained indicate the strength of a function in SD or BD group as compared to control (CD group). *Denotes significant difference as compared to control (*p* < 0.05). **Denotes extremely significant difference as compared to control (*p* < 0.01).

Clearly, at day 1, a majority of functions in the SD group was equivalent or nearly equivalent to that of the CD group in terms of strength (percentiles), but in the BD group, these functions were only 80% or even less (Table 10).

At the end of the test, although a majority of functions in the SD group were still equivalent or nearly equivalent to that of the CD group, all functions have been hugely strengthened in the BD group with statistical significance (Table 10). For example, in the BD group, the following functions were doubled when compared to the CD group, viz., energy production and conversion (231.17%, *p* < 0.05); amino acid transport and metabolism (225.01%, *p* < 0.01); coenzyme transport and metabolism (221.47%, *p* < 0.01); transcription (226.71%, *p* < 0.01); replication, recombination, and repair (259.25%, *p* < 0.01); cell wall/membrane/envelope biogenesis (257.66%, *p* < 0.01); intracellular trafficking, secretion, and vesicular transport (226.26%, *p* < 0.01); secondary metabolites biosynthesis, transport, and catabolism (236.36%, *p* < 0.01); and defense mechanisms (272.40%, *p* < 0.01). More impressively, carbohydrate transport and metabolism function was increased by 4.61-fold (*p* < 0.01) while extracellular structure production function was raised by 305-fold (*p* < 0.01).

## Discussion

Salinity is “one of the main environmental factors that wields a selective pressure on aquatic organisms” (Lamela et al., 2005). A change of salinity may break homeostasis and lead to stress or even death in shrimp (Lamela et al., 2005; Shen et al., 2020). This was demonstrated by Esparza-Leal et al. (2019), who conducted a shrimp bioassay test and discovered that, although shrimp with an initial mean body weight of 3.5 ± 0.7 g could adapt to various salinities (1–35‰) and exhibit no statistically significant differences in growth performance (*p* > 0.05), reduced salinities did affect their physiological and immunological parameters. For instance, shrimp in the 10‰ group showed in hemolymph a higher glucose concentration throughout the 63-day test and a lowest protein content of all treatments at day 30 (*p* < 0.05), both of which indicate stress conditions. This was reflected in their lower survival rate (83.3%) when compared to other groups (93.3% survival rates for groups 1 and 15‰, and 100% for groups 25 and 35‰, *p* < 0.05). They then further revealed that PL at earlier ages, such as PL22, would be more sensitive to the salinity changes than those at older ages as salinity stress tests showed that survival rates were 0% with PL22 and improved progressively with PL37 and PL67 (Esparza-Leal et al., 2020).

Similarly, low salinity is also a stressor for marine bacteria such as *Vibrio parahaemolyticus*, which is pathogenic to human and cultured organisms, and 2.5 and 6‰ salinities were found to be lethal and sublethal for the vibrios, respectively (Huang and Wong, 2012). Therefore, in practice, a strategy of changing salinities, viz., salinity reduction, has currently been applied in some PL production farms in China for the control of vibrios (Liu et al., 2004).

Quite clearly, the effect of salinity reduction treatment on vibrios was evident here as no vibrios at all were found when PL homogenates were spread on TCBS plates. Furthermore, high-throughput sequencing on day-1 PL also confirmed no vibrios in SD1 and BD1, and only 0.05% relative abundance was found in CD1 (Supplementary Table S5). Even these vibrios went undetected or perished at day 7. This finding was also supported in theory by Zhang et al. (2016), who showed that salinity significantly influenced white shrimp microbiota compositions and, thus, biodiversity. As vibrios (and *Vibrionales*) are more adapted to higher salinities than to lower ones (Huang and Wong, 2012; Zhang et al., 2016), they are injured and even perished once subjected to salinity reduction treatment as demonstrated in this test. Nevertheless, our data also illustrate the fact that the current salinity reduction practice doesn’t kill all vibrios outright in some PL and could be improved if a guarantee of zero vibrios in all PL is needed.

Biodiversity is generally recognized as a main determinant of ecosystem functioning (Johnke et al., 2019) and can be quantified via richness (e.g., the number of species), evenness (the relative abundance distribution of those species), or proportional diversity (a combination of richness and evenness, such as the Shannon index, H’; McArt et al., 2012). Of these parameters, the Shannon index was found to outperform species richness as a single variable in explaining variation in disease risk (Chen and Zhou, 2015). In line with this, although there are some exceptions (such as Zheng et al., 2017), it is commonly acknowledged that a healthier and more robust microbial community has a higher biodiversity (and, thus, Shannon index) than an unhealthy/diseased one (e.g., Xiong et al., 2014; Rungrassamee et al., 2016; Chen C.-Y. et al., 2017; Chen W.-Y. et al., 2017; Cornejo-Granados et al., 2017, 2018; Zheng et al., 2017). On reviewing the potentially feasible use of the Shannon index for separating healthy/robust communities from unhealthy/unstable ones, we discover this line hasn’t been drawn yet presently.

Although a meta-analysis study performed by Cornejo-Granados et al. (2018) showed that the Shannon index was estimated to be 1.5–12 for larvae, 1.5–5.6 for postlarvae, 1.0–10 for juveniles, 2–7 for adults of various species of marine shrimp and 1–12 for *Litopenaeus vannemei*, these data are too general and lack discrimination power, especially for a specific stage of a species. Hereby, we reviewed the following papers related specifically to the early stage postlarvae of white shrimp and attempt to draw up a line for the Shannon index for a healthy/robust microbial community for this specific stage (PL7–15).

Zeng et al. (2017) studied healthy white shrimp microbiota at different culture stages in field ponds (salinity not mentioned) from stocking till harvest and found that PL7–8 (0.7 cm in length) had a Shannon index of 1.781. Two weeks after stocking, this index was down to 1.396. Thereafter, indices were raised gradually and peaked at 6.592 and then maintained more or less around 4 with the lowest of 2.458 at one point.

Huang et al. (2016) studied healthy white shrimp microbiota, which were grown indoors, from PL14 (24‰ salinity) to juveniles of 3 months old (27‰ salinity) and showed that the Shannon index was 2.273 in PL14 and 2.187 on average (ranged from 1.255 to 3.218) in juveniles 1–3 months old.

Zheng et al. (2017) studied bacterial communities associated with white shrimp larvae grown at different stages (salinity of 28–32‰) and showed that the Shannon index was 2.1 and 2.37 in healthy PL1 and PL6, respectively, and 2.29 in diseased PL1. If we also consider shrimp at the mysis stage (M1–M3), their Shannon index was above 2 (in the range of 2.11–2.52 in healthy mysis and 2.5 in diseased mysis).

Zhang et al. (2016) studied gut microbiota of white shrimp reared indoors at low (3‰), medium (17‰), and high (30‰) salinities and showed that their Shannon index was 2.40, 2.38, and 1.86, respectively.

Xiong et al. (2019) revealed that the Shannon index was in the range of 3–7 at (post)larval stage and 6–8.5 at the juvenile stage and above 2.5 (2.5–8.5) at the adult stage.

On evaluating these data and taking into consideration that salinity (like Zhang et al., 2016) as well as growth stages (like Huang et al., 2016; Zheng et al., 2017) significantly impact microbiota compositions, we feel it is reasonable to suggest that, for a healthy and robust gut microbiota of white shrimp at the early postlarval stage (PL7–15) a Shannon index of 2.0 or above should be expected; below this value, PL microbiota may not be in a healthy state. This is supported by the better percentage of accumulated weight gain and higher survival rate in the BD group as compared to the SD and CD groups in this study (Table 1). In other words, after salinity reduction treatment, the biodiversities of gut microbiota in the SD and BD groups were reduced to an unhealthy state (Shannon index 0.98, 0.93, respectively), and in the CD group, it was initially still in a relatively healthy state (Shannon index, 2.59) but worsened (Shannon index, 0.94) over the 7-day test period (Table 5). While biodiversity remained at an unhealthy state in the SD group even after the addition of *Bacillus subtilis* (Shannon index, 0.93), it was improved significantly only in the BD group (Shannon index, 7.14) after the addition of BDN-1F2, thus resulting in higher survival rate and better growth (higher percentage accumulated weight gain). This better performance has its solid ground in PL gut microbiota as the predicted functions showed it had higher energy production and conversion function and better defense mechanisms and more (Figure 4 and Table 10).

If postlarvae of other shrimp, such as black tiger shrimp (*Penaeus monodon*), were subject to salinity reduction, the same phenomenon can be observed. This is exactly the case in a study performed by Rungrassamee et al. (2013). In that, Rungrassamee et al. (2013) reported that, when black tiger shrimp PL15 were reared in 15‰ salinity, the Shannon index was 3.46. Once they were transferred to 10–11‰ salinity for further rearing, their Shannon indices dropped to 0.58 and 0.67 in the first (J1) and second (J2) month, respectively and then back to 2.86 in the third month (J3) (most likely after fully adaptation). Even excluding the effect of diets as the same diet was fed to juveniles for 3 months, their Shannon indices were still different. This clearly reflects the impact of salinity reduction on black tiger shrimp gut microbiota as well.

When we further compare microbiota compositions in this study with those of PL15 and juveniles of black tiger shrimp (Rungrassamee et al., 2013), we easily notice a resemblance, viz., the dominance of *Gammaproteobacteria*. In our study (Table 6), the relative abundance of *Gammaproteobacteria* was 99.43, 85.61, and 83.28% in BD1, SD1, and CD1 and 8.44, 99.77, and 99.87% in BD7, SD7, and CD7, correspondingly. In black tiger shrimp (Rungrassamee et al., 2013), its relative abundance was also high, reaching 80.70, 98.50, 98.70, and 89.50% in PL15, J1, J2, and J3, correspondingly.

*Gammaproteobacteria* are generally considered as opportunistic pathogens (Xiong et al., 2015a); they are mostly found to be dominant in diseased shrimp (e.g., Zhu et al., 2016; Chen W.-Y. et al., 2017), such as shrimp with retarded growth (73.7% ± 20.4%), as compared to normal growth (33.3% ± 9.5%) (Xiong et al., 2017). In one case, Xiong et al. (2015b) found that the relative abundance of *Gammaproteobacteria* in shrimp with red or empty intestines (30–40%) was three-to fourfold of that in normal (black) intestines (ca. 10%). Additionally, traditional ecological theory reminds us that such monopoly dominance by one class/genus could render an ecosystem in an extremely unstable state and prone to pathogen invasions (Loreau, 2000) as is the case in this study. Gut microbiota of PL at day 1 as well as of SD7 and CD7 was dominated by *Gammaproteobacteria* (SD7, 99.77%; CD7, 99.89%; Table 7), especially by opportunistic pathogen *Acinetobacter* (SD7, 98.41%; CD7, 98.72%) (Saejung et al., 2014) at the genus level (Supplementary Table S5); we, thus, have reasons to believe that they were not in a healthy/stable state, further affirming the negative effects of salinity reduction on PL shrimp (and too, the black tiger shrimp PL, Rungrassamee et al., 2013).

*Alphaproteobacteria* (such as *Rhodobacterales* and *Rhizobiales*) and *Planctomycetales* are associated with healthy shrimp (Chen W.-Y. et al., 2017; Xiong et al., 2017) and are less abundant in diseased (Zhu et al., 2016; Xiong et al., 2017) or significantly less in slow growth ones (*p* < 0.01) (Fan and Li, 2019). As beneficial bacteria, *Rhodobacteraceae* have been applied in shrimp aquaculture for their abilities to degrade organic compounds in ponds (Huang F. et al., 2018).

While the relative abundance of *Alphaproteobacteria* in SD and CD groups was reduced from 4.8 and 14.02% to 0.08 and 0.04%, respectively, they were increased in the BD group from 0.26 to 12.94% (Table 6). As to the relative abundance of *Planctomycetales*, it was undetected or ≤0.02% in the SD and CD groups. On the contrary, in the BD group, its relative abundance was raised to 3.92% from undetected level (0%) (Table 6). Their increase in abundance should aid PL microbiota health in SD7.

As a group of strong *Proteobacteria* antagonists (Figure 3), *Acidobacteria* was up from an undetected level (0%) to 18.78% in relative abundance in the BD group and down to 0.02% and an undetected level (0%) in the SD and control groups, respectively (Table 6). Digestive enzymes such as amylase, short chain fatty acids such as butyric acid, and immune parameters such as total antioxidant capacity, total nitric oxide synthase, and antibacterial peptide ALF and antibacterial protein Lys were positively correlated with the abundance of *Acidobacteria* (Duan et al., 2020), indicating that it contributes to shrimp digestion and immunity in the BD group and none in the SD and control groups.

*Actinobacteria* is a group of major secondary producers, positively correlated with the immune parameters (such as total antioxidant capacity, total nitric oxide synthase, *HSP70*, *Trx*, *Lys*, *proPO*, *Muc-1*, *Muc-2*, and *Muc-5AC*), indicating that it contributes to the immune response of shrimp (Duan et al., 2020). Again, it was up only in the BD group from being undetected (0%) to 6.64% in relative abundance and down to the undetected level (0%) in both the SD and control groups (Table 6).

*Firmicutes* was up in the BD group only, from 0.12 to 4.92% in relative abundance, but down from 8.43%, 0.15% to both 0.01% in the SD and control groups, respectively (Table 6). *Bacteroidetes* was also up in the BD group only, from 0.06 to 5.03% in relative abundance, and down from 0.2%, 0.55% to both the undetected level (0%) in the SD and control groups, correspondingly (Table 6).

*Firmicutes* and *Bacteroidetes* are also frequently associated with healthy shrimp (Zhang et al., 2014; Huang et al., 2016). *Firmicutes* provides a good index regarding the state of the intestine (Barczynska et al., 2015) as they are significantly more in normal-growth shrimp than in their slow-growth counterparts (*p* < 0.01) (Fan and Li, 2019). Additionally, *Firmicutes* also contributes to the immune response of shrimp (Duan et al., 2020). Further still, *Firmicutes* and *Bacteroidetes* are also involved in fermentation with the former involved in energy harvest and the latter in energy metabolism (Fan and Li, 2019). The *Firmicutes*/*Bacteroidetes* ratio in normal-growth shrimp intestines was 3.08/3.31 (ratio, 0.93), and it was 0.34/6.04 (ratio, 0.06) in slow-growth shrimp intestines (Fan and Li, 2019). This means that energy production and harvest are more balanced in normal-growth shrimp than in slow-growth one. In this study, after salinity reduction treatment, all PL showed unbalanced energy production and harvest (Tables 6, 10) as the ratios below illustrate the point, viz., BD1, 0.12/0.06 (ratio, 2.00), SD1, 8.43/0.2 (ratio, 42.15), and CD1, 0.15/0.57 (ratio, 0.26). Over the 7-day test period, addition of *Bacillus subtilis* did not restore this balance (SD7, 0.01/0), nor the control (CD7, 0.01/0). Only the addition of BDN-1F2 restored this energy production and harvest balance (BD7, 4.92/5.03; ratio, 0.98); as a result, shrimp in this BD group had better growth performance (Table 1).

As another group of very strong *Proteobacteria* antagonists (Figure 3), the phylum *Gemmatimonadetes* is commonly found all over the world in various soil, sediment, and wastewater environments with its relative abundance in soils ranging from 0.2 to 6.5% (DeBruyn et al., 2011). Apart from its ability to accumulate materials such as polyphosphate, a finding on a red-pigmented semiaerobic strain from a freshwater lake in the western Gobi Desert suggests this group of bacteria could commonly have a photoheterotrophic lifestyle (Zeng et al., 2014). This ability of harvest light may provide additional energy for its metabolism and improve the economy of carbon utilization (Zeng et al., 2014). Strangely, Fan and Li (2019) studied *Gemmatimonadetes’* relative abundance in white shrimp intestines and revealed that, as compared to normal-growth shrimp, its abundance in intestines was significantly higher in slow-growth shrimp (*p* < 0.01). This indicates that, even though beneficial bacteria such as *Gemmatimonadetes* are more abundant, if the *Firmicutes*/*Bacteroidetes* ratio is not balanced, shrimp would still not be able to grow well, just as the case of Fan and Li (2019).

In this study, *Gemmatimonadetes* was up in the BD group only from undetected level (0%) to 4.03% in relative abundance but down from 0.01%, 0.02% to the undetected level (0%) in both the SD and control groups, respectively (Table 6). Considering that it could commonly have a photoheterotrophic lifestyle, apart from being a strong *Proteobacteria* antagonist, its abundance surely would provide an added edge for this *Firmicutes*/*Bacteroidetes* ratio-balanced community.

With respect to phylum *Chloroflexi*, order *Anaerolineales* was present only in SD1, CD1, and BD7 with their relative abundances of 0.01, 0.01, and 1.42%, correspondingly (Table 6). As *Anaerolineales* are known heterotrophs capable of metabolizing carbohydrates and peptides (Atashgahi et al., 2015), the increment of *Anaerolineales* in the BD group signifies its enhanced power of carbohydrate and peptide metabolism.

As another group of very strong *Proteobacteria* antagonists (Figure 3), bacteria in the phylum *Verrucomicrobia* have been shown to possess enzymes for the hydrolysis of diverse polysaccharides, including glycoside hydrolases, sulfatases, peptidases, carbohydrate lyases, and esterases (Martinez-Garcia et al., 2012). More recently, a high abundance of *Verrucomicrobia* in the gut microbiota of healthy Chilean subjects, in particular the mucus-degrading bacterium *Akkermansia muciniphila*, was identified (Fujio-Vejar et al., 2017). In this study, *Verrucomicrobia* was initially detected in control (CD1) only, albeit with 0.01% in relative abundance, but had gone undetected at the end of the test (0% in CD7). In the SD group, it was undetected (0%) throughout the test. In the BD group, it came up from undetected (0%) to 0.8% in relative abundance (Table 6) and with *Akkermansia* in its abundance of 0.09% (Supplementary Table S4). Considering that *Akkermansia* has been recently proposed as a hallmark of healthy gut due to its anti-inflammatory and immunostimulant properties (Fujio-Vejar et al., 2017), the increment of *Verrucomicrobia/Akkermansia* would definitely contribute to PL health in BD group.

“*Archaea* is substantial components of complex microbiomes in the environment and in holobionts” and “interact closely with viruses, microorganisms, and holobionts such as plants, animals, and humans” (Moissl-Eichinger et al., 2018). In contrast to Chen W.-Y. et al., 2017, who showed that few archaeal members were in shrimp stomachs after studying the microbiome of white shrimp stomach, we showed that *Archaea* was present in all samples in this study (Table 9). Although their relative abundances went down in the SD and CD groups over the 7-day test period from 0.03% and 0.1% to 0.02% and 0.05%, respectively, it went up in the BD group from 0.02% to 2.0%, a 99-fold increment. As “no archaeal pathogen has been identified thus far” (Moissl-Eichinger et al., 2018) and considering the possible roles of various phyla of *Archaea* existed in PL, viz., *Euryarchaeota* in CH_4_ metabolism (Li et al., 2019), *Thaumarchaeota* in oxidization of ammonia to nitrite (Pan et al., 2018), *Woesearchaeota* in ATP production (Castelle et al., 2015), and in biosynthesis of saccharide molecules that mediate functions from structure and storage to signaling (Breton et al., 2006), and *Crenarchaeota* in carbon assimilation and ammonia oxidation (Hallam et al., 2006) as well as protein degradation (Lloyd et al., 2013), we believe that this vast increment would be beneficial to PL.

From the all-for-the-better changes of microbiota composition at the phylum level discussed above, we are clear that the addition of BDN1-F2 had rectified the unhealthy state of PL, demonstrating its nature as a beneficial agent.

BDN-1F2 was a mutant of wild-type BDN-1 after Co^60^ mutagenesis (data not shown). BDN-1 was identified as a strain of *Bdellovibrionales* (MK159102), closely related to *Bdellovibrionales* strain BDSH06 (EF011103) (Supplementary Figure S1) and characterized as a euryhaline organism that grows at the salinity range of 5–30‰ with optimum at 15‰ (Chen et al., 2019). Considering that white shrimp PL are routinely subjected to a wide range of salinity reduction treatment in practice, it was chosen for this study.

The effect of BDN-1F2 on TCBC, in both PL guts (Table 2) and waters (Table 3), is apparent and statistically significant, both with ca. 3 log reduction (*p* < 0.05). Additionally, Pearson correlation analysis also showed that BDN-1F2 and TCBC in water had a statistically significant correlation with a coefficient at 0.892 (*p* < 0.05) (Supplementary Table S1), indicating the TCBC reduction effect was truly due to BDN1-F2.

Similar findings of bacteria reduction by BALOs in aquaculture have also been reported. For example, Guo et al. (2016) applied BALO strain BDW03 in the rearing of juvenile turbot and revealed that TCBC and TCVC in both waters and intestines were significantly reduced compared to control; Guo et al. (2017) applied BALO strain BDH12 in cold water abalone (*Haliotis discus hannai* Ino) juvenile rearing and showed that, compared to control, TCVC and TCBC in test were reduced by 2.39 and 4.07 log CFU⋅mL^–1^, respectively, in rearing water (*p* < 0.05), and by 3.54 and 4.11 log CFU⋅g^–1^, respectively, in abalone guts (*p* < 0.05). Li et al. (2014) also reported that, by adding BALO strain BDHSH06 to the rearing waters of black tiger shrimp (*Penaeus monodon*) over an 85-day period, TCBC and TCVC were significantly reduced (*p* < 0.05) by 1.3 to 4.5 log CFU mL^–1^ and CFU g^–1^ in both water and shrimp intestines, respectively, compared to control.

To minimize/avoid possible interference of waterborne bacteria in this test, we used boiled and aerated waters to rear PL. Thus, bacteria in rearing water would just be from PL (feces), feed, and the addition of bacteria tested. This should reduce microbial loads in water to low levels and simplify our interpretations. This is exactly the case here as, in the control, there was only ca. 3 log CFU mL^–1^⋅ throughout the test period (Table 3). As such, we did not perform high-throughput sequencing on water samples and just focused on PL guts.

Although Hahn et al. (2017) has excluded *Peredibacter* from the order *Bdellovibrionales*, it was still being classified in this order here according to the existing Ribosomal Database Project (RDP classifier) (see footnote 1). Therefore, by means of high-throughput sequencing, the relative abundance of *Bdellovibrionales* at day 1 in the CD group was 0.01% (Table 6), which was matched by the relative abundance (0.01%) of *Peredibacter* (Table 8). In the SD group, no BALOs were detected throughout the test. And in the BD group, no BALOs were detected at day 1, but at day 7, *Bdellovibrionales* of 0.3% in relative abundance was detected (Table 6). They included *Bdellovibrio* (0.1%), *Micarvibrio* (0%), *Peredibacter* (0.1%), and *Vampirivibrio* (0.08%) (Table 8). This would leave 0.02% in relative abundance for other unclassified BALOs, which implies that BDN-1F2 in the PL gut microbiota would not exceed this figure in abundance even if present. This very few or non-presence of BDN-1F2 in PL guts is in line with the findings of Shatzkes et al. (2017), who were “unable conclusively to detect *B. bacteriovorus* in fecal samples after intrarectally introducing predatory bacteria into the rat gastrointestinal tract,” and of Atterbury et al. (2011), who were “also not able to detect *B. bacteriovorus* in the fecal and cecal contents of young chicks after oral administration of predatory bacteria, signaling that exogenous *B. bacteriovorus* introduced into the gut environment may be short-lived.” Additionally, a study carried out by Guo et al. (2017) also showed that no BDH12 was detected in abalone integer/gut samples even though a concentration of ca. 1 × 10^5^ PFU mL^–1^ was added weekly. Further still, Zeng et al. (2017) also demonstrated that probiotic addition in shrimp culturing did not effectively establish a large population in shrimp intestines with the relative abundance of only 0.04 and 0.002% of *Lactobacillus* and *Bdellovibrio* in shrimp intestines, respectively, and even none in some shrimp.

If we compare BALOs counts in PL guts, derived from using the double-layer agar plating technique (Table 4) with those of high-throughput sequencing (Table 6), they are not matched at day 1 as no BALOs were detected with the latter. A possible explanation for this discrepancy could be that some other microbes that are currently not being recognized as BALOs but could lyse host strain TC exist in PL guts. Alternatively, *Stenotrophomonas* could fill in this gap if it were counted as a BALO as listed by Jurkevitch and Davidov (2007) (Table 8). Unfortunately, isolation of *Stenotrophomonas* was not done and, thus, confirmation could not be performed in this test.

Despite the possible existence of these unrecognized bacteriolytic microbes, the presence of very few or no BDN-1F2 in PL guts at day 7 suggests that PL guts do exert selective pressure on the incoming microbes from its rearing waters (Chen W.-Y. et al., 2017; Zheng et al., 2017; Xiong, 2018; Zhao et al., 2018). Nevertheless, contrary to Zheng et al. (2017), who stated that “bacterial community change in the rearing water only have limited influence on intestine of shrimp larvae,” here our data clearly showed that addition of BDN-1F2 to waters at the start hugely impacted PL gut microbiota, both compositionally (Tables 6–9, Supplementary Tables S4, S5, Figures 1, 2, and Supplementary Figure S2) and functionally (Table 10, Supplementary Tables S2, S3, Figures 3, 4, and Supplementary Figure S3), exceedingly enhanced their biodiversities and strengthened their functionalities. A similar finding, at least compositionally, was demonstrated by Guo et al. (2017), who showed that, at the end of the 90-day test, bacterial communities in abalone guts in test had a higher Shannon diversity index (H’) and Pielou’s evenness index (J’) and a slightly lower richness value (Rs) when compared to control. Moreover, 16S rDNA-PCR and denaturing gradient gel electrophoresis profiles revealed that, of the 27 species/strains sequenced, 51.85% were shared by both control and test and 22.22% reduced, of which 33.33% were *Vibrio*. Finally, they concluded that bacterial communities in test could be slightly more stable and balanced than those in control.

With regards to the mechanism(s) of BALOs exerted on PL gut microbiota, different from the currently existing concept that predatory bacteria prey on the dominant species and, thus, restore the microbial biodiversity in a community but with no further elaborations (e.g., Xiong et al., 2017; Johnke et al., 2019), we here envisage a more elaborate conceptual mechanism on the basis of this study and of Guo et al. (2017); that is, after the introduction of a BALO into a microbial community, it should first attack some of its susceptible microbes on its way, be it dominant or not (of course the BALO would have more chances to meet a dominant at the start, but if the dominant is not to its liking, BALO may not lyse it at all). After a few rounds of multiplication, the BALO has created some space/new niches in the community for some of the preexisting microbes, and, in turn, these microbes, especially those potentially beneficial microbes, would start to grow. At this point, three forces are working hand in hand; that is, while the introduced BALO is still lysing more susceptible microbes, the previous undetected BALOs are starting to show a microbe-lysing effect, and the previous under-representative beneficial microbes (such as Gram-positive) are starting to grow and producing antibacterial substances (against others so as to expand their niches). As these three forces carry on working hand in hand, more and more new niches are being created, and as such, more and more previously under-representative microbes, either beneficial or not so beneficial, gain their niches and start to grow and fill in the newly open and unoccupied niches. Along with the filling of niches, the fourth force, i.e., microbial mutual interactions, be they positive (synergistic) or negative (antagonistic), is kicking in and gradually becomes stronger and stronger, and thus, a new community network is formed and gradually becomes stable. This newly balanced microbial community would be more robust, and functionally more beneficial to its hosts.

The introduced BALO would die off or be kept at low levels as their suitable preys are now below certain levels and/or under the host’s selection pressure.

Applying this conceptual mechanism to this study, we are clear that these four forces are all at work, viz., BDN-1F2, the previously undetected but existed BALOs (Table 8), the previously undetected but existing potential probiotic such as *Actinomyces*, *Bacillus*, *Lactobacillus* (Supplementary Table S4), and pathogens (Supplementary Table S5) as well as positive/negative interactions of bacteria (Supplementary Tables S2, S3, Figures 3, and Supplementary Figure S3).

For example, at the order level (Supplementary Table S2), addition of *Bdellovibrionales* could positively increase the relative abundances of *Bacillales*, *Burkholderiales*, *Nitrospirales*, *Xanthomonadales*, *Desulfuromonadales*, and *Alphaproteobacteria*_incertae_sedis, and so on, and decrease the relative abundances of *Vibrionales*, *Pseudomonadales*, *Rhodobacterales*, *Halobacteriales*, *Gammaproteobacteria*_incertae_sedis, *Aeromonadales*, and the like (*p* < 0.05).

At the genus level (Supplementary Table S3), although the interactions are quite complex, an interesting phenomenon could be noticed, viz., the genera of the same categories (beneficial or harmful) show negative interactions, like probiotic pairs *Bacillus* versus *Actinomyces*, *Lactobacillus* versus *Streptococcus*, and potentially pathogenic pairs *Pseudomonas* versus *Shewanella*, and *Acinetobacter* versus *Klebsiella*. These negative interactions should prevent the overgrowth of the same kind and, thus, maximize the diversity of a whole ecosystem.

All these showed that the addition of *Bdellovibrionales* BDN-1F2 in PL rearing waters has impacted PL gut microbial communities as a whole and restructured its structure and rebuilt its functions.

As new disease outbreaks happen every now and then in aquaculture, such as the acute hepatopancreatic necrosis disease (AHPND) (Chen W.-Y. et al., 2017), the traditional strategy of focusing on a specific pathogen seems not quite adequate and may be outdated from an ecological management perspective. As such, Zhang et al. (2014) pointed out to manage on the entire community level rather than just aiming at vibrios themselves; Guo et al. (2017) also proposed a holistic approach, and De Schryver and Vadstein (2014) also hypothesized that “manipulation of the biodiversity of the gut microbiota can increase the host’s resistance against pathogenic invasion and infection.” Thus, toward this direction, it seems that BALOs are a group of appropriate organisms to be considered for as demonstrated in this study.

## Conclusion

Here in this study, we have shown that gut microbiota of shrimp PL, both compositionally and functionally, have been badly wrecked after being subjected to salinity reduction treatment. Addition of a euryhaline strain of BALOs, BDN-1F2, hugely improved its biodiversity and strengthened its functionalities. On the basis of this study, a Shannon index cutoff value was tentatively suggested so as to differentiate microbiota-healthy PL7-15 from the unhealthy ones. Also, an elaborate conceptual mechanism of BALOs in the rectification/improvement of a microbial community has been proposed.

## Data Availability Statement

The datasets generated for this study can be found in the NCBI Sequence Read Archive (SRA), PRJNA600113.

## Ethics Statement

This study was carried out in accordance with the recommendations of Animal Ethics Committee of Guangdong Province, China.

## Author Contributions

QC has contributed large portion of the work, while the rest minor. JC has designed the experiment and supervising the write-up of the manuscript. All authors contributed to the article and approved the submitted version.

## Conflict of Interest

The authors declare that the research was conducted in the absence of any commercial or financial relationships that could be construed as a potential conflict of interest.

## Footnotes

1 http://rdp.cme.msu.edu/

2 http://www.arb-silva.de/
