# Supplementary material for: BALOs Improved Gut Microbiota Health in Postlarval Shrimp (Litopenaeus vannamei) After Being Subjected to Salinity Reduction Treatment
Source: Front Microbiol. 2020 Jun 30;11:1296. doi: 10.3389/fmicb.2020.01296 (PMC7344170; doi:10.3389/fmicb.2020.01296)
Supplement: Supplementary file 1 [file Data_Sheet_1.zip › Supplementary Figures.pdf]

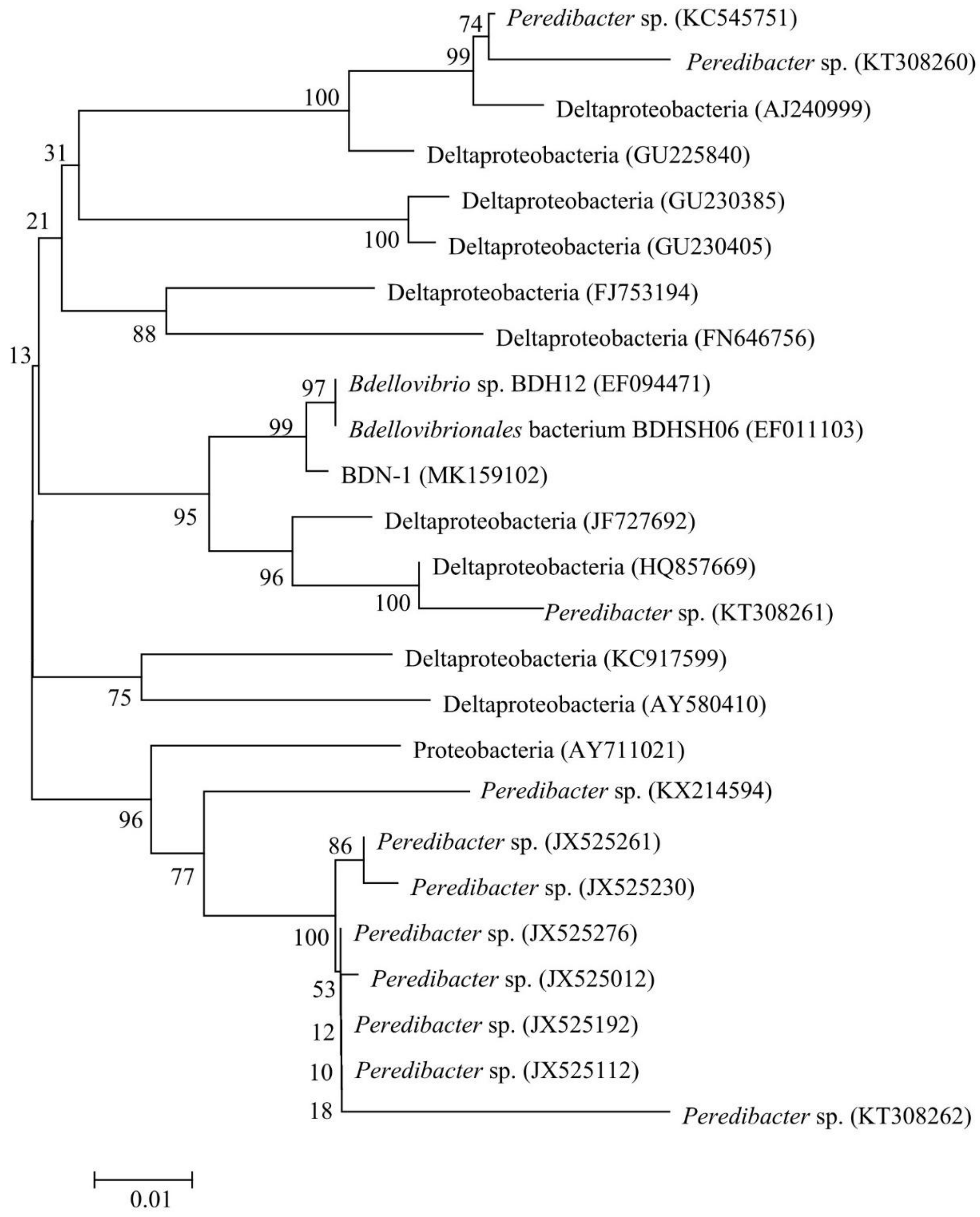

FIGURE S1 A phylogenetic tree depicting the interrelationship between BDN-1 (the wild type of mutant BDN-1F2) and the relevant BALOs based on partial 16S rDNA sequencing analysis. The tree was built with Neighbor-Joining method. Bootstrap was performed with 1000 replicates (Chen et al., 2019).

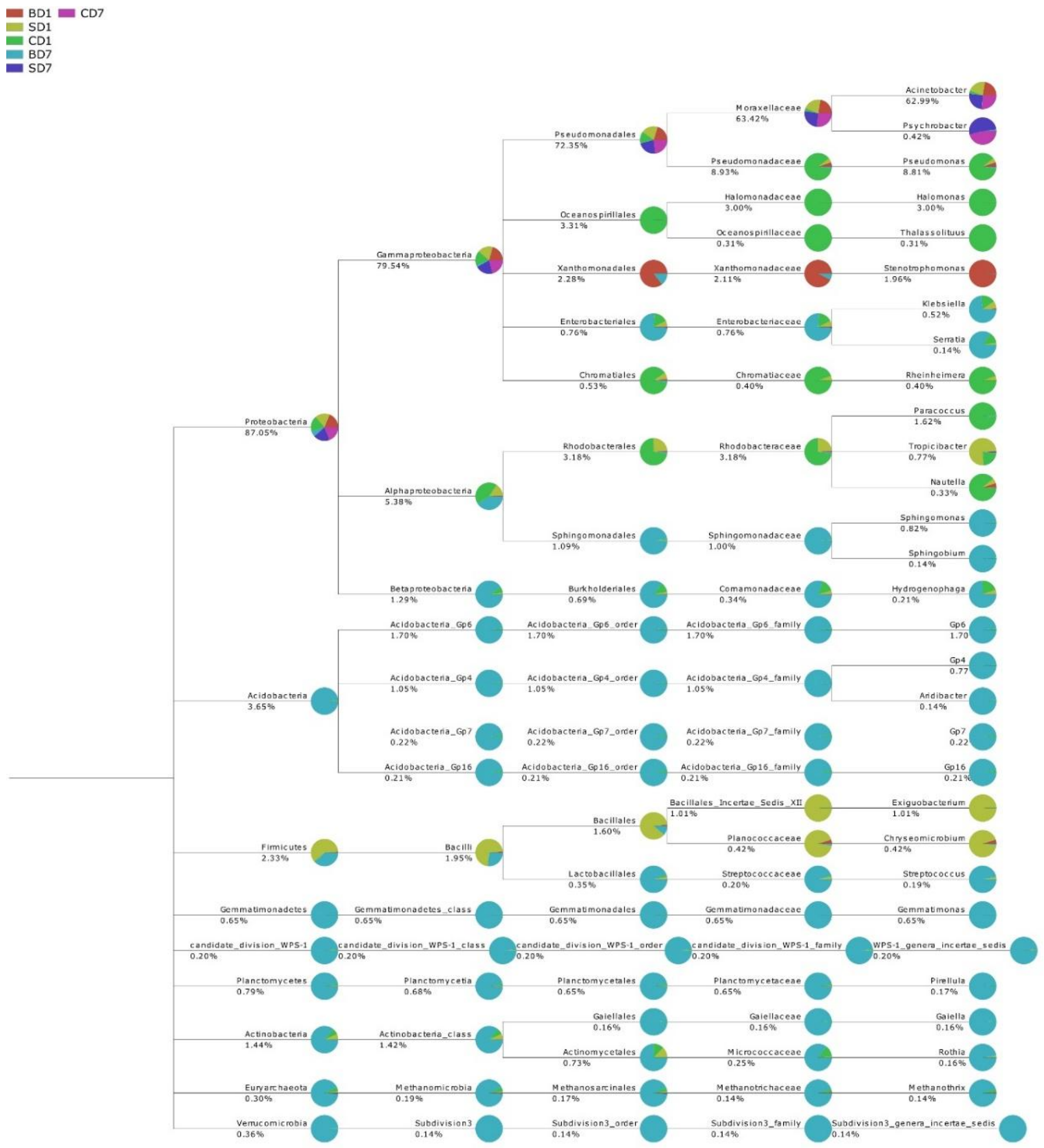

FIGURE S2 Percentages of community abundances at the levels from phylum to order. This taxonomic classification tree was built with ete3 package within the python software. The percentile numbers represent relative abundance in average of that specific taxonomic grouping, while the size of a triangle/pie charts of the hierarchical tree reflects relative abundance of that specific grouping, which was represented in different colours. BD1, SD1 and CD1 represent PL samples at day 1 of test, while BD7, SD7 and CD7 represent PL samples at day 7.

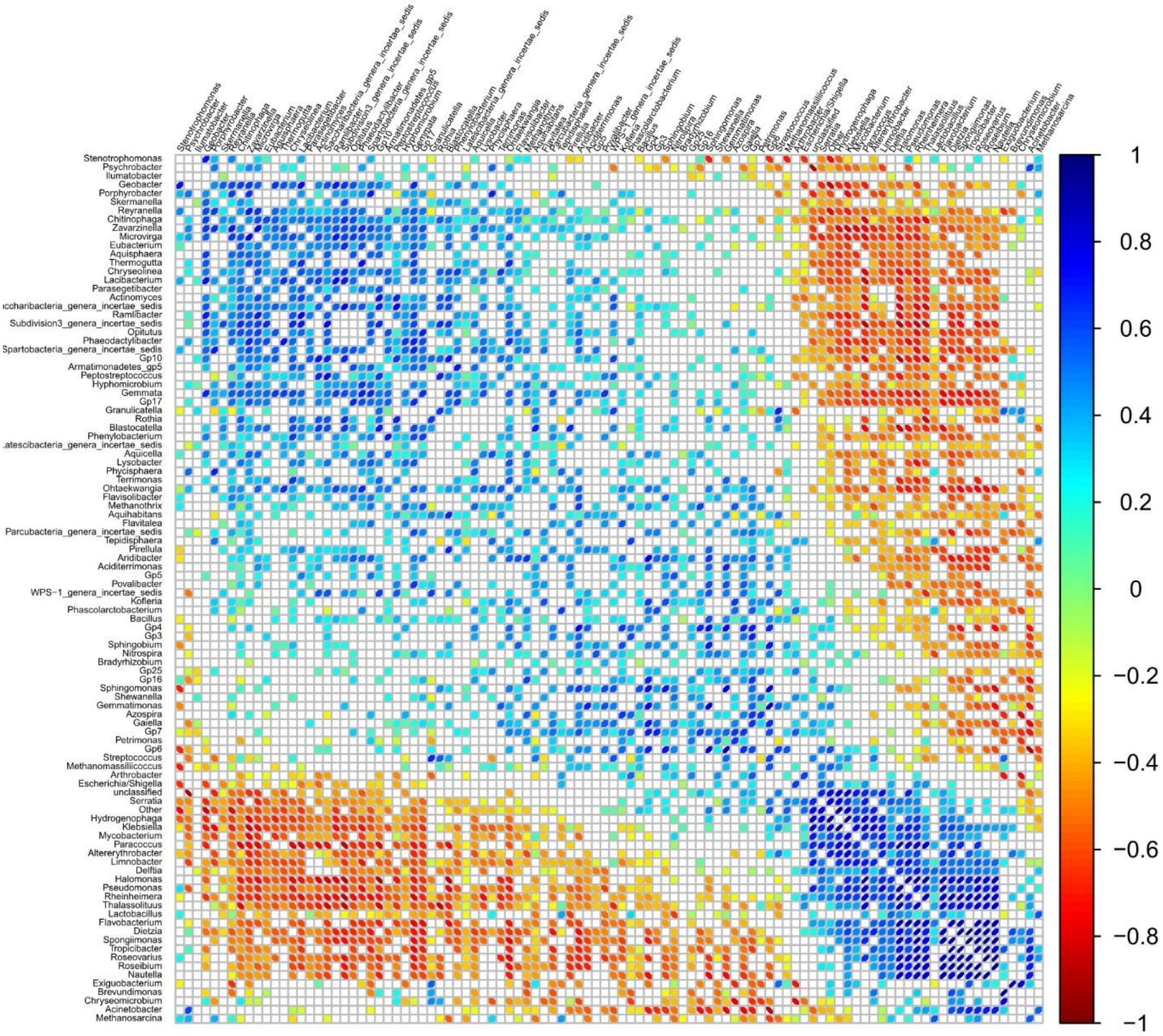

FIGURE S3 Rectangular network interaction graph for microbial communities of BD, SD, and CD groups at the genus level. This graph was drawn only with those data with statistical significance ($p < 0.05$), using SparCC approach (Friedman and Alm, 2012) with kamada kawai algorithm. Each point represents the absolute value of a correlation coefficient. The larger the coefficient value, the smaller the point. Pointing left means positive correlations, pointing right represents negative correlations. Point colours are changed with the right axis colours.
