# Supplementary material for: BALOs Improved Gut Microbiota Health in Postlarval Shrimp (Litopenaeus vannamei) After Being Subjected to Salinity Reduction Treatment
Source: Front Microbiol. 2020 Jun 30;11:1296. doi: 10.3389/fmicb.2020.01296 (PMC7344170; doi:10.3389/fmicb.2020.01296)
Supplement: Supplementary file 1 [file Data_Sheet_1.zip › Supplementary Tables.pdf]

TABLE S1 Pearson Correlation analysis on bacterial and PL growth parameters

	In water			In PL		
	BALOs vs. TCBC	BALOs vs. bodyweight	TCBC (7th d) vs. survival rate	BALOs vs. TCBC	BALOs vs. bodyweight	TCBC (7th d) vs. survival rate
Correlation coefficients	r = 0.892[1]	NC[2]	NC	NC	NC	NC

[1] means significant correlation ($p < 0.05$).

[2] NC: No significant correlation ($p > 0.05$).

TABLE S2 Positive or negative correlations between two orders with statistically significance ($p < 0.05$) [1]

Order	vs. Orders	
	Positive (coefficient ≥ 0.6)	Negative (coefficient ≤ - 0.6)
Aeromonadales	*Gammaproteobacteria*_incertae_sedis, *Halobacteriales, Methanomicrobiales, Nitrososphaerales, Pseudomonadales, Rhodobacterales,*	*Acidimicrobiales, Actinomycetales, Alphaproteobacteria*_incertae_sedis, *Bacteroidales, Bacillales, Bdellovibrionales, Enterobacteriales, Lactobacillales, Methanobacteriales, Myxococcales, Rhizobiales, Rhodospirillales, Sphingomonadales,* Unclassified, *Xanthomonadales,*
Actinomycetales	*Bacillales, Bacteroidales, Bdellovibrionales, Xanthomonadales*	
Bacteroidales	*Alphaproteobacteria*_incertae_sedis, *Bacillales, Bdellovibrionales, Burkholderiales, Desulfuromonadales, Nitrospirales, Xanthomonadales,*	
Bdellovibrionales	*Alphaproteobacteria*_incertae_sedis, *Bacillales, Burkholderiales, Desulfuromonadales, Nitrospirales, Xanthomonadales,*	
*Gammaproteobacteria*_incertae_sedis	*Desulfarculales, Rhodobacterales, Rickettsiales, Thiotrichales, Vibrionales,*	*Actinomycetales, Alphaproteobacteria*_incertae_sedis, *Bacteroidales, Bdellovibrionales, Lactobacillales, Methanosarcinales, Myxococcales, Nitrospirales, Rhizobiales, Rhodospirillales,* Unclassified, *Xanthomonadales*
Halobacteriales	*Nitrosopumilales, Rhodobacterales, Thiotrichales, Vibrionales*	*Actinomycetales, Bacteroidales, Bacillales, Bdellovibrionales, Desulfuromonadales, Enterobacteriales, Myxococcales, Nitrospirales, Rhizobiales, Rhodospirillales, Sphingomonadales,*

		unclassified, *Xanthomonadales,*
Lactobacillales	*Actinomycetales, Alteromonadales, Bacteroidales, Enterobacteriales, Gemmatimonadales, Rhizobiales, Rhodospirillales*	
Methanomicrobiales	*Pasteurellales, Rhodobacterales, Rickettsiales, Vibrionales*	*Actinomycetales, Alphaproteobacteria*_incertae_sedis, *Bacillales, Bacteroidales, Desulfuromonadales, Methanosarcinales, Myxococcales, Nitrospirales, Rhodospirillales, Rhizobiales, Sphingobacteriales, Sphingomonadales,* unclassified, *Xanthomonadales,*
Pseudomonadales	*Rickettsiales, Nitrosopumilales, Vibrionales*	*Actinomycetales, Bacillales, Bacteroidales, Bdellovibrionales, Gemmatimonadales, Nitrospirales, Rhizobiales, Rhodospirillales, Sphingobacteriales, Sphingomonadales,* unclassified, *Xanthomonadales*
Rhodobacterales	*Pseudomonadales, Rickettsiales, Vibrionales*	*Actinomycetales, Bacillales, Bacteroidales, Bdellovibrionales, Methanosarcinales, Myxococcales, Nitrospirales, Rhizobiales, Sphingomonadales, Xanthomonadales*
Rhodospirillales	*Actinomycetales, Alphaproteobacteria*_incertae_sedis, *Bacteroidales, Bdellovibrionales,Enterobacteriales, Methanobacteriales, Myxococcales, Nitrospirales, Rhizobiales, Sphingomonadales, Vibrionales, Xanthomonadales*	
Rickettsiales		*Actinomycetales, Bacillales, Bacteroidales, Gemmatimonadales, Nitrospirales, Rhizobiales, Sphingomonadales, Xanthomonadales*
Sphingomonadales	*Acidimicrobiales, Actinomycetales, Alphaproteobacteria*_incertae_sedis, *Bacteroidales, Bdellovibrionales, Gemmatimonadales, Myxococcales,*	

	Rhizobiales, Sphingobacteriales, Xanthomonadales,	
Vibrionales		*Acidimicrobiales, Actinomycetales, Alphaproteobacteria*_incertae_sedis, *Bacillales, Bacteroidales, Bdellovibrionales, Desulfuromonadales, Myxococcales, Nitrospirales, Rhizobiales, Sphingomonadales, Xanthomonadales,*

[1] Within PL microbiota, potentially positive or negative interactions were performed using SparCC approach (Friedman and Alm, 2012). Only those interactions with statistical significance ($p < 0.05$) and coefficients of 0.6 or higher (absolute values) were given out.

Table S3 Positive or negative correlations between two genera with statistically significance ($p < 0.05$)[1]

Genus	vs. Genera	
	Positive (coefficient ≥ 0.6)	Negative (coefficient ≤ - 0.6)
Aciditerrimonas	*Aridibacter, Gaiella, Gemmatimonas, Gp4, Gp6, Gp16, Kofleria, Rothia, Saccharibacteria*_genera_incertae_sedis, *Sphingomonas*, unclassified,	*Granulicatella*
Acinetobacter	*Devosia, Pseudolabrys,*	*Aciditerrimonas, Gaiella, Gemmata, Gemmatimonas, Gp4, Gp6, Gp7, Gp10, Gp16, Hydrogenophaga, Klebsiella, Lacibacterium, Nitrospira, Pirellula, Rothia, Saccharibacteria*_genera_incertae_sedis, *Sphingobium, Sphingomonas, Streptococcus*, unclassified, WPS-1_genera_incertae_sedis,
Actinomyces	*Aciditerrimonas, Gemmata, Gp6,* unclassified	*Gimesia, Gp10, Ilumatobacter, Nitrospira, Nocardioides*
Arthrobacter	*Povalibacter,*	*Gp4, Gp10, Kofleria,*
Bacillus	*Acinetobacter, Armatimonadetes_gp5, Gp10, Lactobacillus, Methanomassiliicoccus, Methanosarcina, Pseudomonas,*	*Aciditerrimonas, Actinomyces, Arthrobacter, Chryseolinea, Flavitalea, Hydrogenophaga, Hyphomicrobium, Nitrospira, Pirellula, Saccharibacteria*_genera_incertae_sedis, *Shewanella, Sphingobium*
Escherichia/Shigella	*Acinetobacter, Delftia, Gp10, Gp25, Massilia, Nocardioides, Thermogutta*	*Aciditerrimonas, Arthrobacter, Gemmata, Gemmatimonas, Gp3, Gp4, Gp5, Gp6, Hydrogenophaga, Klebsiella, Lacibacterium, Nitrospira, Pirellula, Povalibacter, Sphingobium, Streptococcus*, WPS-1_genera_incertae_sedis
Eubacterium	*Blastocatella, Romboutsia, Saccharibacteria*_genera_incertae_sedis, WPS-1_genera_incertae_sedis,	*Gemmatimonas, Gp4, Gp10, Gp16, Hydrogenophaga, Klebsiella, Microvirga, Mycobacterium, Saccharibacteria*_genera_incertae_sedis, *Shewanella,*

		Sphingomonas,
Flavisolibacter	*Actinomyces, Hydrogenophaga, Phycisphaera, Pseudomonas, Shewanella*	*Gp4, Gp10, Saccharibacteria*_genera_incertae_sedis, *Skermanella*, unclassified
Gemmatimonas	*Aquicella, Aridibacter, Gp4, Gp6 Gp10, Kofleria, Ohtaekwangia, Serratia, Sphingomonas*	
Hydrogenophaga	*Aciditerrimonas, Azospira, Gaiella, Gemmatimonas, Gp6, Gp7, Gp10, Ohtaekwangia, Sphingobium, Streptococcus*	
Klebsiella	*Gp3, Gp4, Kofleria, Ohtaekwangia, Streptococcus,* unclassified	*Methanomassiliicoccus, Petrimonas*
Lactobacillus	*Acinetobacter, Escherichia/Shigella, Gp10, Massilia, Pseudomonas*	*Actinomyces, Arthrobacter, Gemmata, Gemmatimonas Gp3, Gp5, Gp6, Hydrogenophaga, Klebsiella, Nitrospira, Pirellula, Sphingobium, Streptococcus,*
Lysobacter	*Aciditerrimonas, Actinomyces, Chryseolinea, Gemmatimonas, Gp6, Gp10, Massilia, Methanomassiliicoccus*	*Arthrobacter, Gemmata, Gp3, Pirellula, Streptococcus*
Methanosarcina	*Acinetobacter, Escherichia/Shigella, Lactobacillus, Massilia, Methanomassiliicoccus, Pseudomonas, Psychrobacter*	*Aciditerrimonas, Actinomyces, Arthrobacter, Gemmata, Gemmatimonas, Gp3, Gp6, Hydrogenophaga, Klebsiella, Nitrospira, Pirellula, Saccharibacteria*_genera_incertae_sedis, *Sphingobium, Streptococcus*
Nitrospira	*Aciditerrimonas, Gemmata, Gp3, Gp5, Hydrogenophaga, Klebsiella, Povalibacter, Sphingobium,* WPS-1_genera_incertae_sedis,	*Gp10, Kofleria, Methanomassiliicoccus*
Pirellula	*Aciditerrimonas, Gp3, Gp6, Hydrogenophaga, Klebsiella, Lacibacterium, Methanothrix, Mycobacterium, Sphingobium*	*Granulicatella, Methanomassiliicoccus*
Pseudomonas	*Acinetobacter, Aquisphaera, Escherichia/Shigella,*	*Aciditerrimonas, Gemmata, Gemmatimonas, Gp3, Gp4,*

	Massilia, Mesorhizobium, Gp25	*Gp5, Gp6, Gp17, Hydrogenophaga, Shewanella,Streptococcus,*
*Saccharibacteria*_genera_incertae_sedis	*Gp4, Gp6, Gp10, Gp25, Kofleria*	
Shewanella	*Gemmatimonas, Gp5, Kofleria, Ohtaekwangia*	*Aridibacter, Gp25, Methanomassiliicoccus, Methanothrix,*
Sphingobium	*Gaiella, Gemmatimonas, Gp6, Gp7, Gp10, Gp16, Klebsiella, Kofleria, Ohtaekwangia, Rothia, Saccharibacteria*_genera_incertae_sedis, *Sphingomonas, Streptococcus, unclassified*,WPS-1_genera_incertae_sedis,	
Sphingomonas	*Gp4, Gp25, Methanomassiliicoccus, Serratia*	
Streptococcus	*Aciditerrimonas, Gaiella, Gemmatimonas, Gp6, Kofleria, Ohtaekwangia, Rothia, Saccharibacteria*_genera_incertae_sedis, unclassified,	

[1] Within PL microbiota, potentially positive or negative interactions were performed using SparCC approach (Friedman and Alm, 2012). Only those interactions with statistical significance ($p < 0.05$) and coefficients of 0.6 or higher (absolute values) were given out.

TABLE S4 Relative abundance of some functional genera/probiotics and their potential functions

Genus	Relative abundance (%)						Potential functions	Reference
	BD1	SD1	CD1	BD7	SD7	CD7		
Actinomyces	0	0	0	0.38	0	0	Antibacterials producer	Duan et al., 2020
Akkermansia	0	0	0	0.09	0	0	Probiotics	Fujio-Vejar et al., 2017
Arthrobacter	0	0	0.2	0.28	0	0	Bioactives producer	Xue et al., 2016
Bacillus	0	0.01	0.01	0.4	0	0	Probiotics	Zeng et al., 2017
Bacteroides	0	0	0.01	0.11	0	0	Vitamin B_{12} producer	Zeng et al., 2017
Bifidobacterium	0	0	0	0.01	0	0	Probiotics	Ray et al., 2014
Cetobacterium	0	0	0	0.03	0	0	Vitamin B_{12} producer	Zeng et al., 2017
Chryseomicrobium	0.12	2.34	0	0.04	0	0	Protease producer	Farha et al., 2018
Clostridium	0	0	0	0.08	0	0	Commensal-pathogen	Anand et al., 2016
Corynebacterium	0	0.01	0.06	0.05	0	0	Probiotic candidate	Nayak and Mukherjee, 2011
Enterococcus	0	0	0.01	0.1	0	0	Probiotics	Chang and Liu, 2002
Erythrobacter	0	0.04	0.03	0.19	0	0	Shrimp shell degradation	Ruengjitchatchawalya and Wantawin, 2011
Exiguobacterium	0	5.99	0	0.05	0	0	Metabolism of nutrients	Cong et al., 2017
Faecalibacterium	0	0	0.01	0.01	0	0	Shrimp probiotic	Cornejo-Granados et al., 2017

Gemmatimonas	0	0.01	0.02	3.9	0	0	NO_2 metabolism	Cheesanford et al., 2019
Halolactibacillus	0	0	0	0.01	0	0	Antibacterials producer	Suresh et al., 2015
Halomonas	0.01	0.07	17.89	0.01	0	0	Vibrio growth inhibition	Alfiansah et al., 2018
Hydrogenophaga	0	0.07	0.26	0.96	0	0	Organic pollutants degradation	Chung et al., 2007
Lactobacillus	0	0.01	0.03	0.17	0.01	0	Probiotics	Zeng et al., 2017
Lactococcus	0	0	0	0.04	0	0	Probiotics	Dawood et al., 2016
Leuconostoc	0	0	0	0.02	0	0	Probiotics	Martinez and De Martinis, 2006
Massilia	0	0	0	0.13	0	0	Nitrate reduction	Hou et al., 2017
Meridianimaribacter	0	0.01	0.06	0	0	0	Probiotic candidate	Zheng et al., 2017
Micrococcus	0	0	0	0.03	0	0	Anti-*Aeromonas salmonicida* infection	Irianto and Austin, 2002
Paenibacillus	0	0	0	0.01	0	0	Antimicrobial peptides	Abaidullah et al., 2019
Pantoea	0	0	0	0	0.03	0	Disease resistance promotion	Takahashi et al.; 2000
Paracoccus	0	0.09	9.53	0.12	0	0	Dentrification and sulphur oxidation	Jaffer et al., 2019
Propionibacterium	0	0	0	0.02	0	0	Probiotics	Cornejo-Granados et al., 2017
Pseudomonas	2.8	2.75	46.16	1.11	0.01	0.01	Some probiotic, some opportunistic pathogen	Rungrassamee et al., 2013
Pseudorhodobacter	0	0	0	0.02	0	0	Denitrification	Zhou et al., 2019
Psychrobacter	0	0	0.01	0.04	1.31	1.15	Ammonia oxidation	Sangnoi et al., 2016

Rhodobacter	0	0	0.01	0	0	0	Antioxidant activities	An et al., 2019
Ruegeria	0	0.06	0.05	0	0	0	Probiotics	Sonnenschein et al., 2017
Saccharibacteria	0	0	0	0.44	0	0	Shrimp growth enhancer	Fan and Li, 2019
Sphingomonas	0	0.02	0.04	4.83	0.01	0	Vibrio growth inhibition	Yun et al., 2019
Streptococcus	0	0.03	0.01	1.13	0	0	Some probiotic, some opportunistic pathogen	Fan et al., 2019
Streptomyces	0	0	0	0.05	0	0	Antibacterials producer	Mahajan and Balachandran, 2012
Sulfitobacter	0	0.01	0.02	0	0	0	Sulfite–oxidizing ability	Pukall et al., 1999
Tropicibacter	0.03	3.48	1.04	0.03	0.05	0.01	Dentrification and sulphur oxidation	Harwati et al., 2009
Weissella	0	0	0	0	0.01	0	Probiotics	Chen et al., 2017a
SUM	2.97	15.04	75.46	15.11	1.43	1.17		

The relative abundance of potential probiotics in BD group was increased over the 7-day test period, while they were decreased in SD and control (CD) groups.

Table S5 Relative abundance of potential pathogens during testing period and their potential roles

Genus	Relative abundance (%)						Potential roles	Reference
	BD1	SD1	CD1	BD7	SD7	CD7		
Acinetobacter	84.81	82.19	13.12	0.68	98.41	98.72	Opportunistic pathogen	Saejung et al., 2014
Aeromonas	0	0	0.01	0.05	0	0.01	Opportunistic pathogen	Cornejo-Granados et al., 2017
Brucella	0	0	0.01	0.04	0	0	Brucellosis	Mahajan and Balachandran, 2012
Flavobacterium	0.01	0.04	0.13	0.09	0	0	Opportunistic pathogen	Nematollahi et al., 2003
Fusobacterium	0	0	0	0.03	0	0	Commensal-pathogen	Anand et al., 2016
Klebsiella	0.02	0.29	0.54	2.28	0.01	0.01	Pathogen to carp	Das et al., 2018)
Legionella	0	0	0	0.01	0	0	Legionnaires' disease	Hoge and Breiman, 1991
Nautella	0.1	0.12	1.77	0	0	0.01	Pathogen to red alga and brine shrimp	Zheng et al., 2017
Nocardioides	0	0	0	0.13	0	0	Opportunistic pathogen	Lightner and Redman, 1998
Photobacterium	0	0.03	0.01	0	0	0	Opportunistic pathogen/symbiotic	Rungrassamee et al., 2014
Ralstonia	0	0	0	0.04	0	0	Opportunistic human pathogens	Fera et al., 2007
Rickettsia	0	0	0	0.02	0	0	Opportunistic pathogen	Lightner and Redman, 1998

Shewanella	0	0	0.02	0.28	0	0	Shewanellosis	Zhang et al., 2016
Staphylococcus	0	0	0.02	0.08	0	0	Hepatopancreas browned disease in *Eriocheir sinensis*	Fu et al., 2019
Tenacibaculum	0.01	0.03	0.04	0	0	0	“Mouth rot” disease in Atlantic salmon	Jia et al., 2020
Vibrio	0	0	0.05	0	0	0	Vibriosis	Moreno et al., 2017
SUM	84.94	82.66	15.59	3.64	98.42	98.75		

The relative abundance of potential pathogens in BD group was decreased over the 7-day test period, while they were increased in SD and control (CD) groups.
